# Carbazole-Fluorene Copolymers with Various Substituents at the Carbazole Nitrogen: Structure—Properties Relationship

**DOI:** 10.3390/polym15132932

**Published:** 2023-07-03

**Authors:** Věra Cimrová, Drahomír Výprachtický, Aleš Růžička, Veronika Pokorná

**Affiliations:** Institute of Macromolecular Chemistry, Czech Academy of Sciences, Heyrovského nám. 2, 162 00 Prague 6, Czech Republic; vyprachticky@imc.cas.cz (D.V.);

**Keywords:** carbazole-fluorene copolymers, iridium complex, synthesis, photophysics, electrochemistry, electroluminescence

## Abstract

Carbazole derivatives, carbazole-containing polymers and iridium complexes are of interest due to many possible applications in photonics, electronics and biology, particularly as active or hole-transporting layers in organic as well as perovskite devices due to their interesting properties. Here, a series of carbazole-fluorene conjugated copolymers with various substituents at the *N*-carbazole position (2-methoxycarbonylethyl, 2-carboxyethyl, 2-ethylhexyl, and nonan-2,4-dionatoiridium(III)bis(2-phenylpyridine-*N*,*C*^2′^)-9-yl) was prepared by Suzuki coupling. Their photophysical, electrochemical and electroluminescence (EL) properties were studied. Effects of molecular weight and substituents attached to carbazole unit on their properties are reported. The carbazole-fluorene copolymers in dilute solutions exhibited intense photoluminescence (PL) emission in the blue spectral region with high PL quantum yields (78–87%) except for the copolymer with the iridium complex (23%). Similar PL spectra were observed in dilute solutions. More pronounced differences were found in thin film PL and EL properties due to excimer/aggregate formation. Light-emitting devices (LEDs) made of copolymers with 2-ethylhexyl as *N*-carbazole substituent exhibited efficient EL emission with the best performance and the lowest EL onset voltages (3–4 V), while the LEDs made of copolymers with other substituents were not as efficient, but showed anomalous behavior and memory effects in current-voltage characteristics promising also for bio-inspired electronics.

## 1. Introduction

Carbazole-containing polymers, particularly conjugated copolymers with carbazole units incorporated in the polymer backbone are promising due to their properties, especially, photophysical, hole-transporting, photoconductive and electroluminescent, for many applications in photonics and organic electronics, such as light-emitting devices (LEDs), photovoltaic devices (PVDs), field effect transistors (OFETs), sensors, electrochromic devices, etc. [1,2,3,4,5,6,7,8,9,10,11,12]. They can serve not only as active layers in photonic devices, but also as hole-transporting layers for organic and perovskite devices [13,14]. In addition, the polymers with heteroatoms such as nitrogen or oxygen are also useful for hybrid materials in combination with perovskites since they can improve the crystallization morphology, the smoothness and the coverage of the film reducing the voids of the perovskite films during the annealing process and finally, device performances [15,16,17,18]. The empty orbitals of lead or tin make it possible to form the coordination interaction with molecules containing heteroatoms [15] and the hydrogen atom in methylammonium or formamidinium can form hydrogen bonds with atoms with high electronegativity and small radius and therefore, the heteroatom-containing polymers can be used as additives to increase the interaction between grains in perovskite films, thus enhancing the device stability [17]. Furthermore, carbazole derivatives can be used for biological applications, for example, as antiviral agents or as neuroprotective agents with potent antioxidative activity [19,20]. Carbazole units and carbazole-based polymers are also interesting in connection with transition metal complexes. Phosphorescent transition metal complexes have attracted a lot of attention because they can yield both singlet and triplet excitons and consequently, electrophosphorescence can theoretically reach 100% internal quantum efficiency [21]. Among these, iridium(III) complexes are particularly important and the most applicable not only in organic photonic devices, such as LEDs [22,23,24,25], PVDs [26,27,28] and sensors [22,29,30,31,32], but also for many other applications including catalysis [33,34] and biological ones [35]. The complexes can be mixed in polymeric matrix which can be, for example, a carbazole-containing polymer [36]. They can be in the form of phosphorescent iridium(III)-cored dendrimers, where dendrons based on carbazole were also reported [21], or they can be attached or incorporated into the polymer backbone [24,34,37,38], which can improve the processability of active layers in devices.

**Figure 1:**
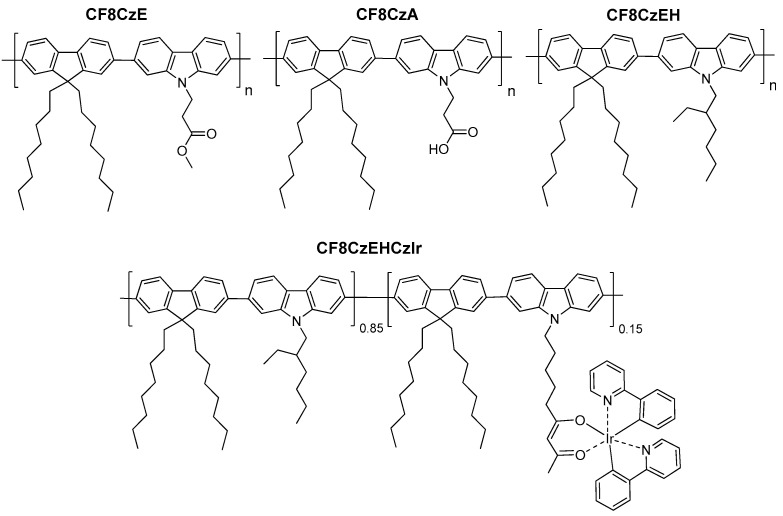
Chemical structure of the fluorene-carbazole-based copolymers under study.

In this paper, we report on syntheses and properties of a series of fluorene-carbazole conjugated copolymers (CF8CzE, CF8CzA, CF8CzEH, and CF8CzEHCzIr) with various *N*-substituents of carbazole units (2-methoxycarbonylethyl, 2-carboxyethyl, 2-ethylhexyl, and nonan-2,4-dionatoiridium(III)bis(2-phenylpyridine-*N*,*C*^2′^)-9-yl) including the synthesis and properties of soluble carbazole units and monomers. Chemical copolymer structures are displayed in Figure 1. The copolymers were synthesized by Suzuki coupling under various conditions yielding copolymers with various molecular weight. Their photophysical, electrochemical and electroluminescence (EL) properties were studied in relation to the structure and molecular weight. Such detailed study gives direct insight into the photophysics, electronic structure and electroluminescence of the fluorene-carbazole copolymers with various *N*-substituted carbazole units including iridium complex substitution. Moreover, investigations of current-voltage characteristics can display additional interesting switching and memory behavior. Many possible applications of carbazole-containing polymers led us to investigate such copolymer series in order to set a firmer basis and to obtain a deeper understanding of relation of the structure and properties.

## 2. Materials and Methods

### 2.1. Materials

9,9-Dioctyl-2,7-bis(1,3,2-dioxaborinan-2-yl)fluorene and 2,7-dibromocarbazole were purchased from Merck (Merck spol. s.r.o., Prague, Czech Republic). 9-(2-Ethylhexyl)-2,7-dibromocarbazole was synthesized according to our previous paper [39]. Tetrahydrofuran (THF, Lach-Ner, Ltd., Neratovice, Czech Republic) was refluxed (7 h) with LiAlH_4_ and distilled. 1,2-Dichlorobenzene (DCB) was purchased from Acros Organics. The iridium trichloride hydrate was purchased from TCI (TCI Europe, N.V., Zwijndrecht, Belgium). The Silicagel 60 (0.063–0.200 mm, Merck) was used for column chromatography (columns Ø 3–4 cm × 60 cm). The TLC was performed with Silicagel 60 F_254_ aluminium sheets (Merck) and the *R*_F_ values have the common meaning (*R*_F_ = distance traveled by substance/distance traveled by solvent front).

### 2.2. Methods

Average molecular weights (*M*_w_, *M*_n,_) of the copolymers were determined by size exclusion chromatography (SEC) measurements using pump Deltachrom (Watrex, s.r.o., Prague, Czech Republic), autosampler Midas, with two columns PL gel MIXED-C LS, particle size 5 μm. Evaporative light scattering detector PL-ELS-1000 (Polymer Laboratories) was used; tetrahydrofuran (THF) or 1,2-dichlorobenzene (DCB) was the mobile phase. Polystyrene standards were used for calibration.

Thin polymer films were prepared by spin-coating from toluene, THF or DCB solutions in a glove box under nitrogen atmosphere (M. Braun Inertgas-Systeme GmbH, Garsching, Germany). The films were spin-coated onto fused silica substrates for optical measurements, onto indium-tin oxide (ITO) substrates covered with a thin layer of poly [3,4-(ethylenedioxy)thiophene]/poly(styrenesulfonate) (PEDOT:PSS) for LED preparation or coated on Pt wire electrode by dipping for electrochemical measurements. The 25 nm thick PEDOT:PSS layers were prepared by spin-coating and dried in vacuum at 396 K for 15 min prior to the polymer film deposition. Polymer films were dried in vacuum (10^−3^ Pa) at 353 K for 2 h. In addition, 20 nm thick calcium (Ca) covered with 60–80 nm thick aluminum (Al) electrodes were vacuum-evaporated on the top of polymer films to form LED devices. Typical active area of the LEDs was 4 mm^2^.

UV-vis spectra were measured on a Perkin-Elmer Lambda 35 UV-VIS spectrometer and photoluminescence (PL) spectra using Perkin-Elmer LS55 (PerkinElmer Instruments, Shelton, CT, USA) and FS5 Fluorescence spectrophotometer with SC-30 Integrating sphere module (Edinburgh Instruments Ltd., Livingston, UK). Electroluminescence (EL) spectra were recorded using a Spectra Pro 300i monochromator/spectrograph (Acton Research Corporation, Acton, MA, USA) with a single photon-counting detection (SPEX, RCA C31034 photomultiplier). LEDs were supplied from a Keithley 237 source measure unit (Keithley Instruments, Inc., Cleveland, OH, USA), which served for simultaneous recording of the current flowing through the sample. Current–voltage and EL intensity–voltage characteristics were recorded simultaneously using the Keithley 237 source measure unit and a silicon photodiode with integrated amplifier HUV-4000B (EG&G, Montgomeryville, PA, USA) for the detection of total light output or luminance meter Minolta LS110 (Minolta Camera Co., Ltd., Osaka, Japan). A voltage signal from the photodiode was recorded with an Agilent 34401A multimeter (Agilent Technologies, Inc., Santa Clara, CA, USA). EL measurements were performed under nitrogen atmosphere. Absorption spectra of thin films were also recorded in the glove box using fiber optics connected to the spectrophotometer and also under ambient laboratory atmosphere. Layer thicknesses were measured using a KLA-Tencor P-10 or P-17 profilometer (KLA-Tencor Corporation, Milpitas, CA, USA). Cyclic voltammetry (CV) was performed with a PA4 polarographic analyzer (Laboratory Instruments, Prague, Czech Republic) with a three-electrode cell. Platinum (Pt) wire electrodes were used as both working and counter electrodes, and non-aqueous Ag/Ag^+^ electrode (Ag in 0.1 M AgNO_3_ solution) was used as the reference electrode. CV measurements were made in solution of 0.1 M tetrabutylammonium hexafluorophosphate (TBAPF_6_) in anhydrous acetonitrile in nitrogen atmosphere. Ferrocene (Fc/Fc+) were used as an external standard; therefore, its CV curves were also recorded. Typical scan rates were 20, 50 and 100 mV s^−1^.

### 2.3. Syntheses of Polymers

#### 2.3.1. Poly[9,9-dioctylfluorene-2,7-diyl-*alt*-9-(2-methoxycarbonylethyl)carbazole-2,7-diyl]s (CF8CzE-1, CF8CzE-2, CF8CzE-3)

##### CF8CzE-1

The methyl 3-(2,7-dibromocarbazol-9-yl)propionate (0.6317 g, 1.537 mmol) and 9,9-dioctyl-2,7-bis(1,3,2-dioxaborinan-2-yl)fluorene (0.8581 g, 1.537 mmol) were weighted into a glass reactor equipped with a septum, efficient stirrer, argon inlet and reflux condenser. The reactor was degassed in repeating argon/vacuum cycles (6×) and purged with argon (30 min). The tetrakis(triphenylphosphine)palladium(0) (0.035 g, 1 mol% per monomers) in degassed xylene (35 mL) and the degassed aqueous 15% NaHCO_3_ (35 mL) were added gradually via septum and the reaction mixture was stirred at 90 °C for 64 h under a gentle stream of argon. The reaction was end-capped first with addition of phenylboronic acid pinacol ester (0.2061 g, 1.03 mmol, 3 h at 90 °C) and second with 2-ethylhexyl bromide (0.5 mL, 2.81 mmol, 4 h at 90 °C). After cooling, the reaction mixture was poured into methanol (700 mL) and a green precipitate was filtered off (S2) and dried (yield: 2.22 g > 100% contains inorganic material). The raw material was purified by extraction (Soxhlet) with THF (150 mL) and after volume reduction (30 mL), the yellow fluorescent THF solution was precipitated into methanol (700 mL). The yellow fluorescent polymer CF8CzE-1 was filtered off (S2) and dried (yield: 0.7912 g, 82%). *M*_w_ = 18,500, *M*_n_ = 8400, *Ð* = 2.2 (SEC in THF). 

^1^H NMR (300.13 MHz, THF-*d*_8_, 330 K, δ): 8.25–7.10 (m, 12 H, aromatic H), 4.86 (br s, 2H, N–CH_2_), 3.60 (s, 3H, OCH_3_), 2.97 (br s, 2H, N–C–CH_2_), 2.22 (br s, 4H, 2 × fluorene-CH_2_), 1.30–0.85 (m, 24H, 12 × CH_2_), 0.78 (s, 6H, 2 × CH_3_); (found: sum of aromatic integrals Σ_arom_ = 18.82, sum of aliphatic integrals Σ_aliph_ = 65.83, Σ_arom_/Σ_aliph_ = 0.29; calcd. = 0.29); (Figure S1). ^13^C NMR (75.45 MHz, THF-*d*_8_, 330 K, δ): 172.4 (C=O), 152.7, 142.6, 142.3, 141.2, 140.8, 127.4, 123.4, 122.9, 121.2, 120.8, 120.0, 108.4 (all aromatic C), 56.4 (fluorene C), 51.8, 41.3, 39.7, 34.2, 34.0, 32.9, 32.7, 31.1, 30.1, 23.4, 14.3 (all aliphatic C); (Figure S2). FTIR (ATR): 3019, 2923, 2851, 1736, 1602, 1563, 1453, 1434, 1359, 1326, 1255, 1202, 1169, 1062, 996, 933, 854, 802, 757, 661, 590, 567, cm^−1^; (Figure S3).

##### CF8CzE-2

The 9,9-dioctyl-2,7-bis(1,3,2-dioxaborinan-2-yl)fluorene (1.3569 g, 2.43 mmol) and methyl 3-(2,7-dibromocarbazole-9-yl)propionate (1 g, 2.43 mmol) were weighted into a glass reactor equipped with a septum, efficient stirrer, argon inlet and reflux condenser. The reactor was degassed in repeating argon/vacuum cycles (6×) and purged with argon (30 min). The degassed xylene (25 mL), the aqueous 13% NaOH (25 mL) and the tetrakis(triphenylphosphine)palladium(0) (0.056 g, 1 mol% per monomers) were added via septum into the reactor. The reaction mixture was refluxed at 145 °C for 60 min under a gentle stream of argon. After cooling, the reaction mixture was precipitated into methanol/water (800/100 mL). The raw CF8CzE-2 was filtered off, washed with water, and dried. The polymer was purified by precipitation from THF into methanol/water (800/100 mL). The yellow-green precipitate of CF8CzE-2 was filtered off (S4) and dried (yield: 1.34 g, 86%). *M*_w_ = 12,310, *M*_n_ = 6040, *Ð* = 2.04 (SEC in DCB).

^1^H NMR (300.13 MHz, THF-*d*_8_, 330 K, δ): 8.25–7.10 (m, 12 H, aromatic H), 5.00–4.65 (m, 2H, N–CH_2_), 3.60 (s, 3H, OCH_3_), 3.05–2.80 (m, 2H, N–C–CH_2_), 2.26 (br s, 4H, 2 × fluorene-CH_2_), 1.40–0.80 (m, 24H, 12 × CH_2_), 0.80–0.65 (m, 6H, 2 × CH_3_); (Figure S4). ^13^C NMR (75.45 MHz, THF-*d*_8_, 296 K, δ): 172.5 (C=O), 152.5, 142.1, 142.0, 141.1, 140.5, 127.4, 123.2, 122.9, 121.2, 120.9, 120.0, 108.3 (all aromatic C), 56.2 (fluorene C), 51.8, 41.3, 39.4, 34.2, 34.0, 32.8, 31.1, 30.7, 30.2, 23.5, 14.4 (all aliphatic C); (Figure S5). FTIR (ATR): 3028, 2923, 2851, 1736, 1601, 1563, 1453, 1434, 1359, 1326, 1255, 1203, 1170, 1063, 996, 933, 854, 802, 757, 722, 590, 567, cm^−1^; (Figure S6).

##### CF8CzE-3

The 9,9-dioctyl-2,7-bis(1,3,2-dioxaborinan-2-yl)fluorene (1.3569 g, 2.43 mmol) and methyl 3-(2,7-dibromocarbazole-9-yl)propionate (1 g, 2.43 mmol) were weighted into a glass reactor equipped with a septum, efficient stirrer, argon inlet and reflux condenser. The reactor was degassed in repeating argon/vacuum cycles (6×) and purged with argon (30 min). Monomers were dissolved in degassed xylene (22 mL). The aqueous 15% NaHCO_3_ (25 mL) and tetrakis(triphenylphosphine)palladium(0) (0.056 g, 1 mol% per monomers) in degassed xylene (3 mL) were added via septum and the reaction mixture was refluxed at 145 °C for 80 min under a gentle stream of argon. The reaction was end-capped first with addition of phenylboronic acid pinacol ester (0.2061 g, 1.03 mmol, 40 min at 145 °C) and, second with 2-ethylhexyl bromide (0.67 mL, 3.78 mmol, 30 min at 145 °C). After cooling, the reaction mixture was poured into methanol/water (700/100 mL) and the precipitate was filtered off and dried (yield: 2.40 g). The raw CF8CzE-3 was dissolved in THF, filtered (S4), and reprecipitated into methanol. The purified orange CF8CzE-3 was filtered off (S4) and dried (yield: 1.31 g, 84%). *M*_w_ = 6270, *M*_n_ = 3240, *Ð* = 1.94 (SEC in DCB).

^1^H NMR (300.13 MHz, THF-*d*_8_, 330 K, δ): 8.25–6.62 (m, 12 H, aromatic H), 5.00–4.65 (m, 2H, N–CH_2_), 3.60 (s, 3H, OCH_3_), 3.05–2.80 (m, 2H, N–C–CH_2_), 2.23 (br s, 4H, 2 × fluorene-CH_2_), 1.35–0.85 (m, 24H, 12 × CH_2_), 0.85–0.65 (m, 6H, 2 × CH_3_); (found: sum of aromatic integrals Σ_arom_ = 15.29, sum of aliphatic integrals Σ_aliph_ = 50.39, Σ_arom_/Σ_aliph_ = 0.30; calcd. = 0.29); (Figure S7). ^13^C NMR (75.45 MHz, THF-*d*_8_, 330 K, δ): 172.4 (C=O), 152.7, 142.6, 142.2, 141.2, 140.8, 127.5, 123.4, 122.9, 121.2, 120.8, 120.0, 108.4 (all aromatic C), 56.4 (fluorene C), 51.8, 41.4, 39.7, 34.2, 34.0, 32.7, 31.1, 30.6, 30.1, 23.4, 14.3 (all aliphatic C); (Figure S8). FTIR (ATR): 3024, 2924, 2852, 1735, 1602, 1562, 1453, 1434, 1358, 1327, 1255, 1203, 1170, 1063, 996, 933, 854, 802, 757, 698, 590, 568, cm^−1^; (Figure S9).

#### 2.3.2. Poly[9,9-dioctylfluorene-2,7-diyl-*alt*-9-(2-carboxyethyl)carbazole-2,7-diyl] (CF8CzA)

The polymer CF8CzE-1 (0.30 g) was dissolved in THF (30 mL), 20 mL of 10% NaOH in methanol/water (3:1 *v*/*v*) was added and the reaction mixture was refluxed (85 °C) for 5 h. After cooling, the reaction mixture was poured into 1% NaOH (500 mL) and mixed 24 h. The slightly turbid solution was filtered (S3) and acidified with HCl (1:1 *v*/*v*) to pH = 2–3. Gradually, a nice cloudy yellow polymeric acid CF8CzA precipitated out of the solution. The CF8CzA was filtered off (S3) and dried. Yield: 0.2802 g (96%). The hydrolysis of methyl ester groups –COOCH_3_ to carboxylic acid groups –COOH was confirmed by ^1^H NMR and FTIR spectroscopies. 

^1^H NMR (300.13 MHz, THF-*d*_8_, 296 K, δ): 8.25–7.10 (m, 12 H, aromatic H), 4.85 (br s, 2H, N–CH_2_), 2.94 (br s, 2H, N–C–CH_2_), 2.19 (br s, 4H, 2 × fluorene-CH_2_), 1.30–0.85 (m, 24H, 12 × CH_2_), 0.78 (s, 6H, 2×CH_3_); (found: sum of aromatic integrals Σ_arom_ = 11.29, sum of aliphatic integrals Σ_aliph_ = 35.54, Σ_arom_/Σ_aliph_ = 0.32; calcd. = 0.32); (Figure S10). FTIR (ATR): 3025, 2925, 2853, 1713, 1604, 1566, 1454, 1434, 1360, 1328, 1236, 1135, 1060, 997, 935, 855, 803, 758, 660, 590, 569, cm^−1^; (Figure S11).

#### 2.3.3. Poly[9,9-dioctylfluorene-2,7-diyl-*alt*-9-(2-ethylhexyl)carbazole-2,7-diyl]s (CF8CzEH, CF8CzEH-1, CF8CzEH-2) 

The 9-(2-ethylhexyl)-2,7-dibromocarbazole (0.4372 g, 1.00 mmol) and 9,9-dioctyl-2,7-bis(1,3,2-dioxaborinan-2-yl)fluorene (0.5584 g, 1.00 mmol) were weighted into a glass reactor equipped with a septum, efficient stirrer, argon inlet and reflux condenser. The reactor was degassed in repeating argon/vacuum cycles (6×) and purged with argon (30 min). The tetrakis(triphenylphosphine)palladium(0) (0.023 g, 1 mol% per monomers) in degassed xylene (20 mL), the degassed aqueous 15% NaHCO_3_ (20 mL) and Aliquat 336 (20 mg) were added gradually via septum and the reaction mixture was stirred at reflux (150 °C) for 90 min under a gentle stream of argon. The reaction was end-capped first with addition of phenylboronic acid pinacol ester (0.1 g, 0.49 mmol, 30 min at 150 °C) and, second with 2-ethylhexyl bromide (0.3 mL, 1.68 mmol, 1 h at 150 °C). After cooling, the upper xylene (dark) layer was separated from the water (clear) layer and precipitated into methanol (600 mL). The bluish polymer precipitate of CF8CzEH was filtered off (S3) and dried (yield: 0.6325 g, 95%). The raw CF8CzEH was extracted (stirred) with toluene/xylene (60 mL, 2:1 *v*/*v*) at room temperature for 24 h and the insoluble part was filtered off (S3) and dried (yield: 0.4525 g). The toluene/xylene filtrate was reduced (15 mL) and precipitated into methanol (600 mL). The fine precipitate was filtered off (S4) and dried as CF8CzEH-1 (yield: 0.1256 g, 19%). The toluene/xylene insoluble part was dissolved in DCB (20 mL) and precipitated into methanol (700 mL) to get after mixing (24 h) a fine gray luminescent material, which was filtered off (S4) and dried as CF8CzEH-2 (yield: 0.3895 g, 58%). CF8CzEH-1: *M*_w_ = 36,080, *M*_n_ = 20,680, *Ð* = 1.74 and CF8CzEH-2: *M*_w_ = 49,360, *M*_n_ = 34,120, *Ð* = 1.45 (SEC in DCB). Both materials exhibited the same NMR spectra and the same FTIR spectra.

^1^H NMR (300.13 MHz, CDCl_3_, 296 K, δ): 8.30–7.30 (m, 12H, aromatic H), 4.33 (br s, 2H, N–CH_2_, 4.33 (br s,), 2.35–1.85 (m, 5H, 2 × fluorene-CH_2_ + >CH–C–N), 1.70–0.85 (m, 32H, 16 × CH_2_), 0.78 (t, *J* = 9.0 Hz, 12H, 4 × CH_3_); (sum of aromatic integrals: Σ_arom_ = 6.211, sum of aliphatic integrals: Σ_aliph_ = 27.646; Σ_arom_/Σ_aliph_ = 0.23; calcd. = 0.24); (Figure S12). ^13^C NMR (75.45 MHz, CDCl_3_, 296 K, δ): 151.9, 142.2, 141.1, 140.1, 139.6, 126.5, 122.0, 121.8, 120.4, 119.3, 118.8, 107.6 (all aromatic C), 55.4, 47.4, 40.6, 39.7, 31.9, 31.3, 30.2, 29.8, 29.4, 28.9, 24.6, 24.5, 24.1, 23.2, 22.7, 14.1, 11.1 (all aliphatic C); (Figure S13). FTIR (ATR): 2954, 2924, 2852, 1602, 1452, 1414, 1328, 1252, 1192, 1134, 998, 854, 802, 758, 746, 724, cm^−1^; (Figure S14).

#### 2.3.4. Poly{9-[nonan-2,4-dionatoiridium(III)bis(2-phenylpyridine-N,C^2′^)-9-yl]carbazole- 2,7-diyl-co-9,9-dioctylfluorene-2,7-diyl-*co*-9-(2-ethylhexyl)carbazole-2,7-diyl} (CF8CzEHCzIr)

The 9-(2,7-dibromocarbazol-9-yl)nonan-2,4-dionato-bis(2-phenylpyridine-*C*^2^,*N*’)-iridium(III) complex (0.1960 g, 0.2 mmol), 9,9-dioctyl-2,7-bis(1,3,2-dioxaborinan-2-yl)fluorene (0.5584 g, 1.0 mmol), and the 9-(2-ethylhexyl)-2,7-dibromocarbazole (0.3498 g, 0.8 mmol) were weighted into a glass reactor equipped with a septum, efficient stirrer, argon inlet and reflux condenser. The reactor was degassed in repeating argon/vacuum cycles (6×) and purged with argon (30 min). The tetrakis(triphenylphosphine)palladium(0) (0.023 g, 1 mol% per monomers) in degassed xylene (20 mL) was added via septum and the reaction mixture was stirred (100 °C, 30 min) under argon to remove any dissolved gasses. Then, the aqueous 15% NaHCO_3_ (20 mL) and Aliquat 336 (20 mg) were added via septum and the reaction mixture was refluxed at 150 °C for 90 min under a gentle stream of argon. The reaction was end-capped first with addition of phenylboronic acid pinacol ester (0.1 g, 0.5 mmol, 30 min) and, second with 2-ethylhexyl bromide (0.3 mL, 1.68 mmol, 30 min). After cooling, the lower water/carbonate layer was wasted and the upper organic layer was diluted with xylene (5 mL) and precipitated into methanol (800 mL). The precipitate was filtered off, dried and reprecipitated from xylene (20 mL, filtered S3) into methanol (700 mL). The yellow precipitate was filtered off and dried. Yield: 0.6977 g (90%). Anal. Calcd for [(C_43_H_36_N_3_O_2_Ir)_0.15_-(C_29_H_40_)_1.0_-(C_20_H_23_N)_0.85_](747.27): C, 84.30; H, 8.78; N, 2.44. Found: C, 84.25; H, 8.80; N, 2.45. *M*_w_ = 16,730, *M*_n_ = 9240, *Ð* = 1.81 (SEC in DCB).

^1^H NMR (300.13 MHz, CDCl_3_, 296 K, δ): 8.50–6.22 (m, aromatic H), 5.15 (s, –(O)C=CH–C(O)–), 4.33 (br s, N–CH_2_), 2.30–1.90 (m, fluorene-CH_2_, –(O)C–CH_2_), 1.71 (s, –(O)C–CH_3_), 1.60–1.00 (m, CH_2_), 0.99–0.70 (m, CH_3_); (sum of aromatic integrals: Σ_arom_ = 7.823, sum of aliphatic integrals: Σ_aliph_ = 26.836; Σ_arom_/Σ_aliph_ = 0.29; calcd. = 0.28); (Figure S15). ^13^C NMR (75.45 MHz, CDCl_3_, 296 K, δ): 187.3, 185.0, 168.8, 168.7, 151.9, 151.5, 151.1, 148.0, 144.7, 142.2, 142.1, 141.7, 141.1, 140.9, 140.3, 140.0, 139.6, 128.9, 127.6, 126.5, 123.9, 122.0, 121.3, 120.6, 120.1, 118.8, 107.6, 100.2 (all aromatic C), 55.4, 55.2, 47.3, 40.6, 39.7, 31.9, 31.3, 30.2, 29.3, 28.9, 26.7, 24.5, 24.1, 23.2, 22.7, 14.1, 11.1 (all aliphatic C); (Figure S16). FTIR (ATR): 2952, 2924, 2854, 1604, 1580, 1454, 1436, 1328, 1252, 1192, 1136, 1062, 998, 854, 826, 802, 754, 728, 670, cm^−1^; (Figure S17).

## 3. Results and Discussion

### 3.1. Syntheses of Monomers

**Scheme 1:**
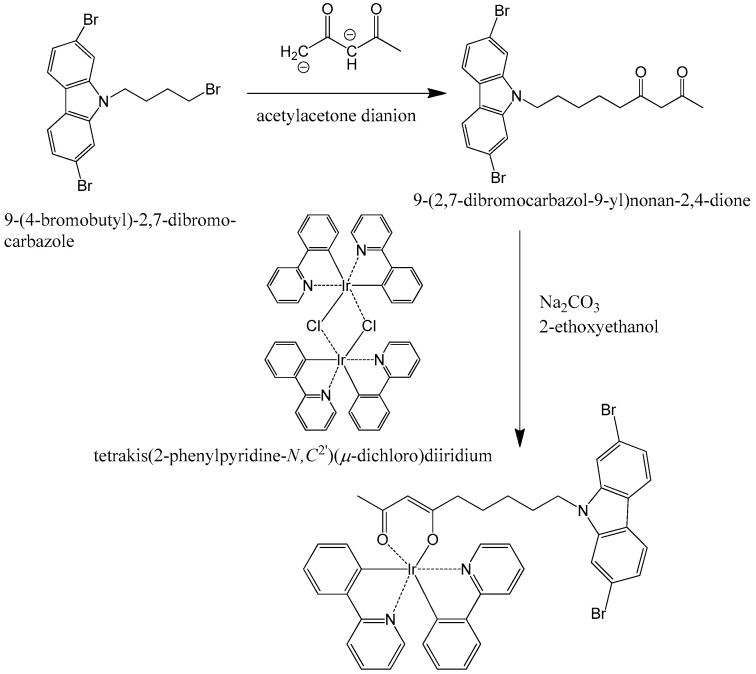
Synthesis of 2,7-dibromo-9-[nonan-2,4-dionatoiridium(III)bis(2-phenylpyridine-*N,C*^2′^)-9-yl]carbazole monomer.

The 9-(2-ethylhexyl)-2,7-dibromocarbazole was synthesized as described in detail in our previous paper [39] and 9,9-dioctyl-2,7-bis(1,3,2-dioxaborinan-2-yl)fluorene was a commercial product. The methyl 3-(2,7-dibromocarbazole-9-yl)propionate was prepared by nucleophilic addition of 2,7-dibromocarbazole to methyl acrylate. The 2,7-dibromo-9-[nonan-2,4-dionatoiridium(III)-bis(2-phenylpyridine-*N*,*C*^2′^)-9-yl]carbazole was synthesized via a multistep procedure shown in Scheme 1. Monomer syntheses are thoroughly described in our previous paper [40] and in Supporting Information, where their characterization including NMR and FTIR spectra (Figures S18–S26) is presented.

### 3.2. Syntheses of Polymers

**Scheme 2:**
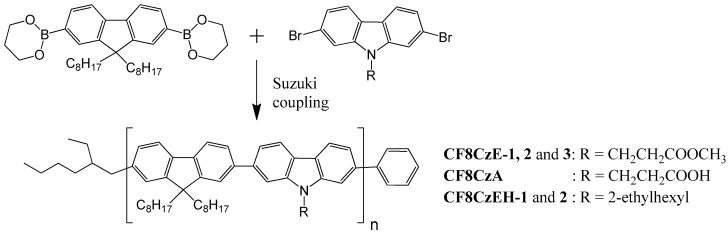
Synthesis of fluorene-carbazole copolymers by Suzuki coupling.

The fluorene-carbazole copolymers were synthesized by Suzuki coupling of the 9,9-dioctyl-2,7-bis(1,3,2-dioxaborinan-2-yl)fluorene with two carbazole comonomers: a) the methyl 3-(2,7-dibromocarbazol-9-yl)propionate (CzE-Br) or b) 9-(2-ethylhexyl)-2,7-dibromocarbazole (CzEH-Br) as it is depicted in Scheme 2. The poly[9,9-dioctylfluorene-2,7-diyl-*alt*-9-(2-methoxycarbonylethyl)carbazole-2,7-diyl]s (CF8CzE-1, 2 and 3) were synthesized in xylene/aq.base solutions under different reaction conditions (temperature, time, Table 1) yielding the copolymers with various average molecular weights (*M*_w_, *M*_n_, Table 2). The synthesis at higher temperature was investigated intentionally. The high-temperature short-time Suzuki coupling was successfully demonstrated for the syntheses of some donor-acceptor copolymers in xylene, chlorobenzene and dichlorobenzene yielding copolymers with higher molecular weight and low dispersity than those from long-term low-temperature syntheses as reported in our previous paper [41]. However, the copolymers CF8CzE-1, 2 and 3 follow the other way. The Suzuki coupling performed at 90 °C for 64 h gave higher *M*_w_, *M*_n_ (CF8CzE-1) than the reaction performed at 145 °C for 1.33 h yielding the copolymer CF8CzE-3 with lower molecular weight. Using the strong base (xylene/aq.NaOH), copolymer CF8CzE-2 with a nearly two-times-higher molecular weight than that of CF8CzE-3 was gained in an even shorter time of 1 h. In the case of CF8CzE-2, the system with the strong base was applied intentionally to synthesize the polyacid CF8CzA in one pot by Suzuki coupling and ester hydrolysis. Unfortunately, the ^1^H NMR and FTIR analyses (–COOCH_3_ signals) revealed the CF8CzE-2 as only one product. On the other hand, the polyacid CF8CzA was successfully prepared by alkaline hydrolysis of CF8CzE-1 in THF/methanol/aq.NaOH solution. Here, the hydrolysis of methyl ester groups (–COOCH_3_) to carboxylic acid groups (–COOH) was quantitatively confirmed by ^1^H NMR and FTIR spectroscopies. The alkaline ester hydrolysis took place in a homogeneous system unlike the heterogeneous one.

The poly[9,9-dioctylfluorene-2,7-diyl-*alt*-9-(2-ethylhexyl)carbazole-2,7-diyl] was prepared by Suzuki reaction in xylene at a short time of 1 h. The reaction proceeded smoothly and yielded the copolymer CF8CzEH with high molecular weight. The copolymers CF8CzEH-1 and CF8CzEH-2 with various average molecular weight (Table 1 and Table 2) were prepared by fractional precipitation of CF8CzEH raw material from toluene/xylene and from 1,2-dichlorobenzene into methanol, respectively. All the prepared fluorene-carbazole copolymers were characterized by ^1^H and ^13^C NMR and FTIR spectroscopies, which results confirmed the copolymer structure, yields as summarized in Experimental and shown in Supporting Information Figures S1–S14, and by SEC, which results are displayed in Table 2.

**Scheme 3:**
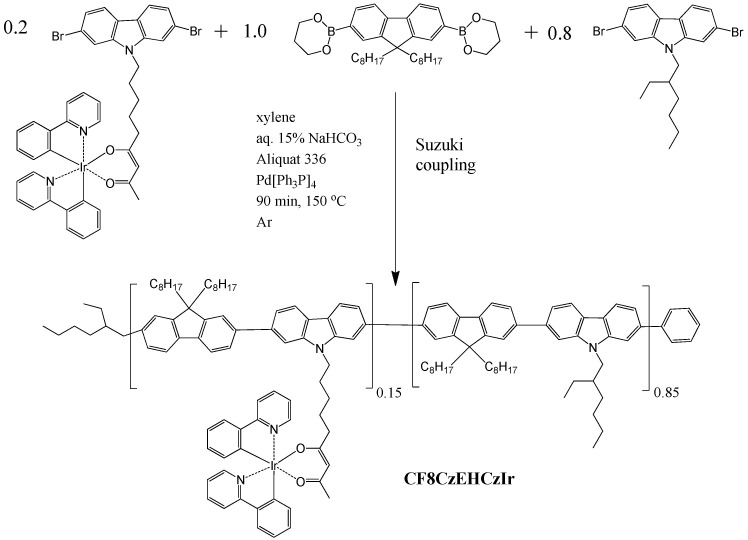
Synthesis of copolymer CF8CzEHCzIr by Suzuki coupling (the composition [(C_43_H_36_N_3_O_2_Ir)_0.15_-(C_29_H_40_)_1.0_-(C_20_H_23_N)_0.85_] was determined by elemental analysis (ex N).

The copolymer poly{9-[nonan-2,4-dionatoiridium(III)bis(2-phenylpyridine-*N*,*C*^2′^)-9-yl]carbazole-2,7-diyl-*co*-9,9-dioctylfluorene-2,7-diyl-*co*-9-(2-ethylhexyl)carbazole-2,7-diyl} (CF8CzEHCzIr) containing iridium complex was synthesized by Suzuki coupling of the 2,7-dibromo-9-[nonan-2,4-dionatoiridium(III)bis(2-phenylpyridine-*N*,*C*^2′^)-9-yl]carbazole (CzIr-Br), 9,9-dioctyl-2,7-bis(1,3,2-dioxaborinan-2-yl)fluorene, and 9-(2-ethylhexyl)-2,7-dibromocarbazole comonomers as it is shown in Scheme 3. The CF8CzEHCzIr was characterized by ^1^H and ^13^C NMR and FTIR spectroscopies, yield, elemental analysis and by SEC (see Experimental and Supporting Information Figures S15–S17, and Table 2).

### 3.3. Photophysical Properties

**Figure 2:**
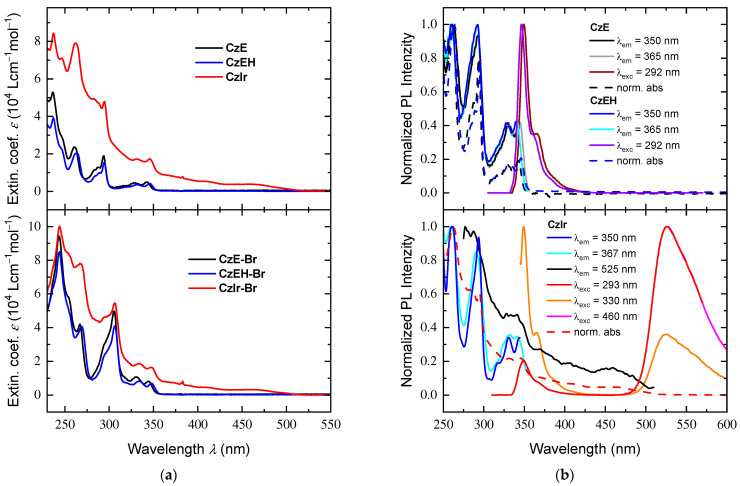
(**a**) UV-vis absorption spectra of the carbazole CzE, CzEH, CzIr units and the CzE-Br, CzEH-Br, CzIr-Br monomers measured in dilute THF solutions. (**b**) Photoluminescent spectra of the carbazole CzE, CzEH, CzIr units measured in dilute THF solutions.

Photophysical properties of carbazole-containing copolymers were studied in solutions and in thin films. The absorption spectra of the *N*-substituted carbazole units methyl 3-(carbazole-9-yl)propionate (CzE), 9-(2-ethylhexyl)carbazole (CzEH), 9-[nonan-2,4-dionatoiridium(III)-bis(2-phenylpyridine-*N*,*C*^2′^)-9-yl]carbazole (CzIr), and monomers (CzE-Br, CzEH-Br, CzIr-Br) were also measured in tetrahydrofuran (THF) solutions for comparison. An example of these spectra is displayed in Figure 2a. The absorption spectra of carbazole units are well resolved with maxima listed in Table 3. The absorption maxima for CzE are slightly blue shifted compared to those for CzEH and CzIr. The spectral features are characteristic of the *N*-substituted carbazoles [42,43,44,45,46]. The (0,0) transitions to the first and second excited singlet states (at 346 and 295 nm, respectively, for CzEH) are polarized along the short in-plane and long in-plane molecular axes respectively. For the π-electron transition in a carbazole planar molecule of C_2v_ symmetry, the allowed transitions are short axis and long axis polarized transitions. The blue shift in absorption CzE spectra can be interpreted in terms of an interaction between transition dipoles localized on carbazole and the ester substituent. The CzIr spectrum shows that the attachment of iridium complex to carbazole led to an increase in the extinction coefficient in the UV region, which can be ascribed to a π-π* transition centered on the phenylpyridine cyclometalated ligands, and in the extended absorption up to 500 nm due to singlet−triplet metal to ligand charge transfer (MLCT) transitions induced by heavy element effect. The brominating of the carbazole units led to red shifts of the absorption maxima in the (CzE-Br, CzEH-Br, CzIr-Br) monomer spectra compared with the corresponding units, namely, of the second transition polarized along long in-plane molecular axis.

The PL emission spectra of the *N*-substituted carbazole units are displayed in Figure 2b and their PL maxima summarized in Table 3. CzE and CzEH exhibited maximum at 347 and 349 nm, respectively. The shape of the spectra is similar for various excitation wavelengths. A blue shift is observed for CzE compared with CzEH similarly as in absorption spectra. The excitation spectra followed well the absorption spectra. The PL emission spectrum of CzIr consists of two main peaks, whose mutual contributions depended on the excitation wavelength. The blue emission originates from the carbazole unit and the emission at 525 nm corresponds to the electronic transition of a mixed intraligand/metal to ligand charge transfer character (ILCT/MLCT). The excitation spectra for the emission wavelengths of 350–370 nm, i.e., corresponding to PL of the carbazole unit, are similar to the excitation spectra of the CzEH unit, whereas the excitation spectrum for the emission wavelength of 525 nm corresponding to the MLCT transition differs. It follows more the absorption spectrum of CzIr.

**Figure 3:**
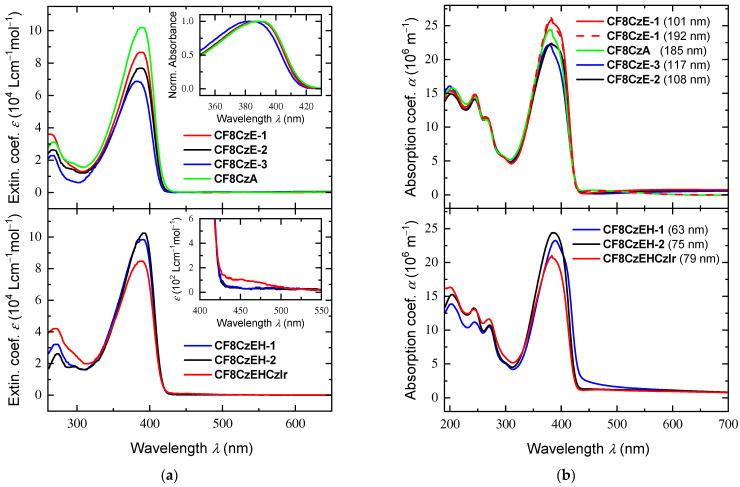
UV-vis absorption spectra of copolymers under study measured (**a**) in dilute THF solutions and (**b**) in thin films.

The absorption spectra of the copolymers measured in THF solutions displayed in Figure 3a exhibit a broad band absorption with a maximum ranging from 380 to 392 nm corresponding to the π-π^*^ transition of conjugated backbone.

The maximum position and extinction coefficient values correlate well with the copolymer molecular weight. CF8CzE-3, with the lowest molecular weight (*M*_w_ = 6270), exhibited an absorption maximum at the shortest wavelength of 383 nm, which is blue-shifted by ca. 5–7 nm compared to the wavelength of the absorption maxima of the other copolymers CF8CzE-1 and CF8CzE-2 with *M*_w_ > 12000. The detail of normalized spectra maximum is shown in the inset. The maximum of CF8CzEH-2 located at 392 nm is also red shifted compared with that of CF8CzEH-1 with lower molecular weight. The values of extinction coefficients in maximum are in the range from 7–10 × 10^4^ L mol^−1^ cm^−1^. An increase in the extinction coefficient with increasing molecular weight was observed for both CF8CzE and CF8CzEH copolymers. Incorporation of Ir-complex lead to the long wavelength absorption extension up to 500 nm corresponding to the Ir-complex absorption (see inset in Figure 3a).

The absorption spectra of the thin films displayed in Figure 3b are similar to the corresponding spectra measured in their THF solution. The long-wavelength absorption maxima located at 378–383 and 383–389 nm in CF8CzE and CF8CzEH films, respectively, are slightly blue-shifted compared with the corresponding maxima in the solution spectra (see Table 4 and Table 5), which indicate H-like aggregate formation in the solid state [47].

**Figure 4:**
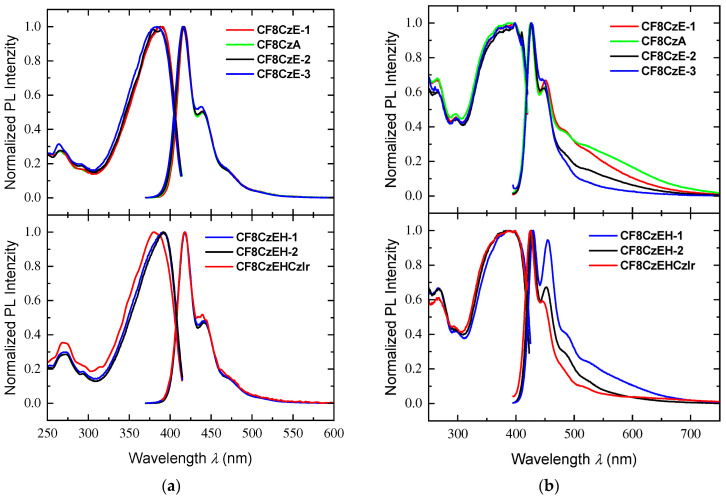
Normalized PL excitation (*λ*_PLem_ at *λ*_PLmax_) and emission (*λ*_PLexc_ at *λ*_absmax_ nm) spectra of copolymers under study measured (**a**) in dilute THF solutions and (**b**) in thin films.

The copolymers exhibited blue photoluminescence in both solution and thin films. The PL excitation and emission spectra measured in the THF solutions are displayed in Figure 4a, and the characteristic data are summarized in Table 4. Emission spectra measured in solutions are similar for all copolymers under study. They exhibit a vibration structure with a main maximum at 416–417 and 418–419 nm for CF8CzE and CF8CzEH, respectively, which can be assigned to the 0-0 singlet transition, and an additional maxima and shoulders, which correspond to 0-1 and 0-2 transitions, without any strong dependence on molecular weight. The main maximum in CF8CzA spectrum at 423 nm is slightly red shifted compared with that of CF8CzE, which could reflect solvatochromic behavior as proved by the PL spectra measured in chloroform, where the maxima in both CF8CzE and CF8CzA spectra were located at 416 nm. The PL spectrum of CF8CzEHCzIr is similar to the CF8CzEH spectra with a slight difference in the 0-0 and 0-1 transition contributions. Excitation spectra follow well the absorption ones. The CF8CzE copolymers in THF exhibited high PL quantum yields of 75–78%. Higher values of 87 and 81% were measured for CF8CzEH-1 and CF8CzEH-2, respectively. Incorporation of Ir-complex lead to more pronounced decrease in PL quantum yield by factor more than 3.5. The quantum yield in CF8CzEHCzIr solution was 23% only. The results indicate that the substitution in *N*-position of carbazole unit influence the both maximum wavelength position and quantum yield value.

PL spectra of thin films are displayed in Figure 4b and the characteristic data are summarized in Table 4. For the thin films, the emission maxima are red-shifted by approximately 15 nm compared with the maxima in corresponding THF solutions, which indicates aggregate formation in the solid state as already mentioned above. For CF8CzE and CF8CzEH films, the main maxima are located at about 430 and 433 nm, respectively. Compared to solution PL spectra, more pronounced differences in the shapes of thin film PL spectra for copolymers with different molecular weight were observed, which could be related to the differences in aggregate formation due to different molecular weight. It should be noted that the spectrum shape can also slightly differ for different thicknesses. The relative PL efficiency of a thin film, *Φ*_PLrel_, was introduced to compare the PL efficiencies of the copolymers under study. Similar *Φ*_PLrel_ values were evaluated for CF8CzE-1, CF8CzEH-1 and CF8CzEH-2 films. The *Φ*_PLrel_ values of CF8CzE-2 and CF8CzE-3 films were nearly two and five time lower than that of CF8CzE-1 films, respectively, i.e., *Φ*_PLrel_ decreases with the decreasing molecular weight. The *Φ*_PLrel_ differences could be explained by differences in the molecular arrangement in thin films. The most significant decrease was observed for CF8CzEHCzIr films, where the *Φ*_PLrel_ value decreases by factor 20 compared with that of CF8CzEH-1 or CF8CzEH-2. The PL quenching in CF8CzEHCzIr films is more pronounced than in its solutions, which indicates more pronounced excimer and/or aggregate formation in the solid state for the CF8CzEHCzIr copolymer with Ir complex than for the CF8CzEH copolymer.

### 3.4. Electrochemical Properties

**Figure 5:**
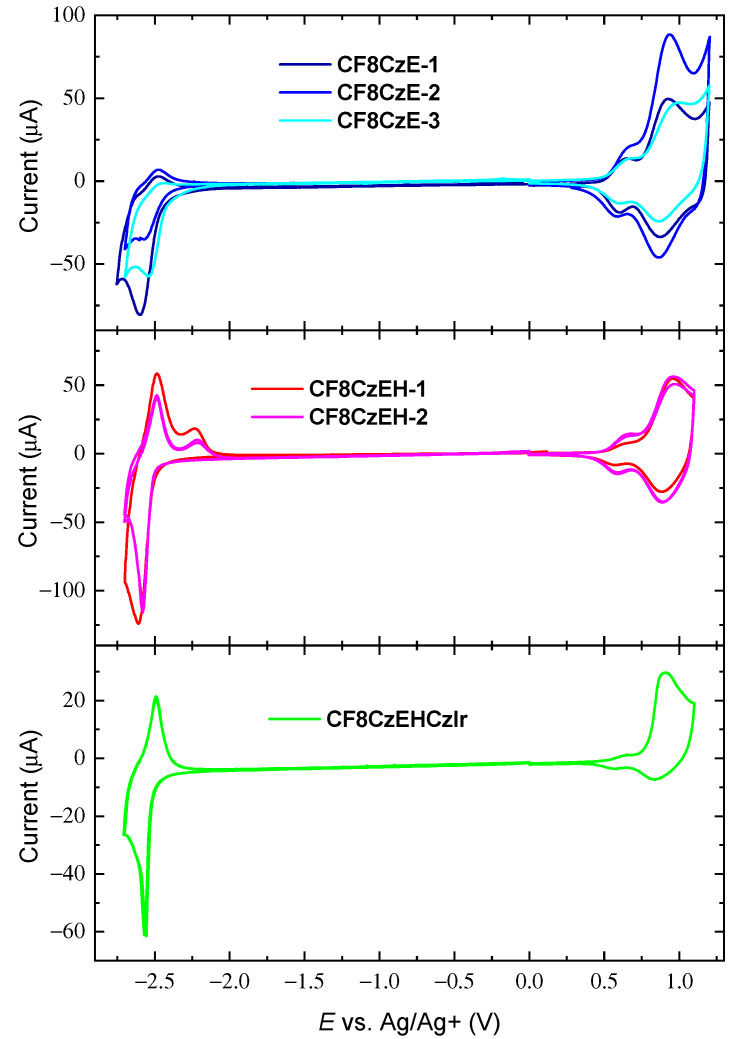
Representative cyclic voltammograms of copolymer thin films coated on Pt wire.

Information about the electronic structure of the copolymers under study was obtained by means of cyclic voltammetry (CV) measurements. Representative CV curves of copolymer thin films coated on a Pt wire are displayed in Figure 5. Quasi-reversibility in the oxidation and reduction was observed. The CF8CzEH and CF8CzEHCzIr copolymers exhibit a better reversibility in the reduction than the CF8CzE copolymers. In oxidation, two pairs of peaks occurring at different potentials indicated the presence of two oxidation reactions for all copolymers under study. The peak current of the first oxidation peak was notably less than that of the second oxidation peak. Similarly, when the potential was reversed, the first de-doping is a higher peak than the second de-doping peak, that occurred upon decreasing the potential further. The ionization potential (HOMO level), *E*_IP_, and electron affinity (LUMO level), *E*_A_, were evaluated from the onset potentials, *E*_onset_, of the oxidation and reduction peaks using the equation *E*_IP_ (*E*_A_) = |– (*E*_onset_ − *E*_ferr_) − 4.8| eV, where *E*_ferr_ is the half-wave potential for ferrocene vs. the Ag/Ag^+^ electrode and 4.8 eV below the vacuum level is the reference energy level of ferrocene, which was used as an internal standard. The *E*_IP_ and *E*_A_ values given in Table 6 were obtained as averages from CV curves measured at a scan rate of 50 mVs^−1^. Similar *E*_IP_ and *E*_A_ values of 5.2–5.3 eV (evaluated from the first oxidation onsets) and 2.2–2.3 eV, respectively, were found for all copolymers. The electrochemical bandgaps, *E*_g_^elc^, of 2.93–3.04 eV evaluated from the first oxidation onsets are in a good agreement with the optical bandgap values, *E*_g_^opt^, of 2.94–2.96 eV determined from the absorption spectra of thin films.

### 3.5. Electroluminescence

**Figure 6:**
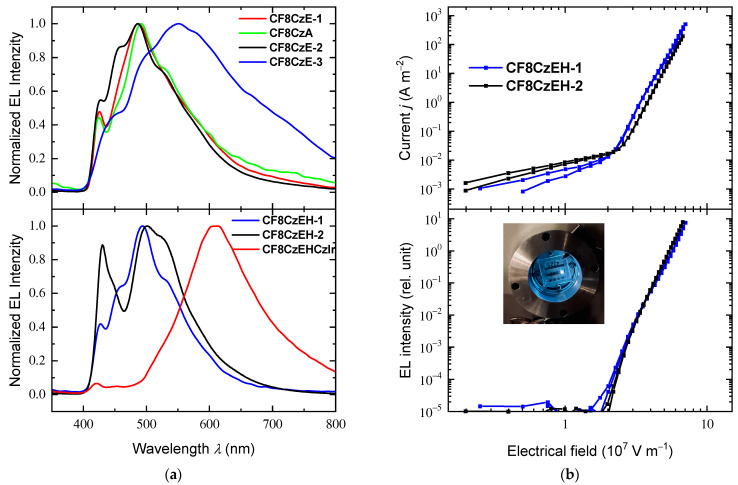
(**a**) Normalized EL spectra of the ITO/PEDOT:PSS/copolymer/Ca/Al LEDs and (**b**) dependence of current density and EL intensity on applied electrical field of the ITO/PEDOT:PSS/copolymer/Ca/Al LEDs made of CF8CzEH-1 and CF8CzEH-2 (picture in Inset) with thicker active layers.

The copolymers were tested as an active layer in LEDs. LEDs with a hole-injecting electrode formed by ITO covered with a PEDOT:PSS layer and an electron-injecting electrode of calcium covered with aluminum (Ca/Al) were prepared and studied. The electroluminescence (EL) spectra of the LEDs are shown in Figure 6a.

The CF8CzE-1 LED exhibited a dominant blue-greenish EL emission with a maximum at 487 nm with a smaller contribution of the blue emission with a maximum at 426 nm, which dominates in PL spectra of thin films. Similar features were observed in the EL spectra of CF8CzA and CF8CzE-2 with the main maxima at 487 and 486 nm, respectively. The EL spectra of LEDs made of CF8CzE-3 copolymer with the lowest molecular weight show a maximum at 551 nm, which is significantly red-shifted compared to PL and also to the EL spectra of the CF8CzE-1, CF8CzA and CF8CzE-2 LEDs. The EL spectra of CF8CzEH-1 and CF8CzEH-2 LEDs exhibited dominant emission bands with maxima at 494 and 502 nm, respectively, and also contributions in blue spectral region with maxima at ca. 430 nm, where the thin film PL dominate. The contribution of the blue emission was higher in the EL spectra of CF8CzEH-2 LEDs than in the spectra of LEDs made of the CF8CzEH-1 with the lower molecular weight. There are also some differences in the EL spectra for the different active layer thicknesses. The EL spectral shapes could be influenced by resonant effect, which depends on the active layer thickness [48,49,50]. A higher contribution of the emission in blue spectral region was observed for thinner active layers. Spectral changes could be further influenced by differences in film morphology–molecular arrangement. During the LED operation, the contribution of blue emission decreased. This effect could be connected with the molecular re-arrangement into the parallel oriented molecular segments as was observed in LED based on the poly(1,4-phenylene)s [51,52]. The EL maximum of the CF8CzEHCzIr LED (at 615 nm) was significantly red shifted compared to the EL spectra of the CF8CzEH-1 and CF8CzEH-2 LEDs and compared with the CF8CzEHCzIr thin-film PL maximum. The blue emission is nearly fully suppressed in this case. The red shifts of the EL maxima compared with the PL ones indicate that charge trapping plays a significant role in the EL process and the differences in the spectra indicate that different centers dominate in the EL and PL processes.

**Figure 7:**
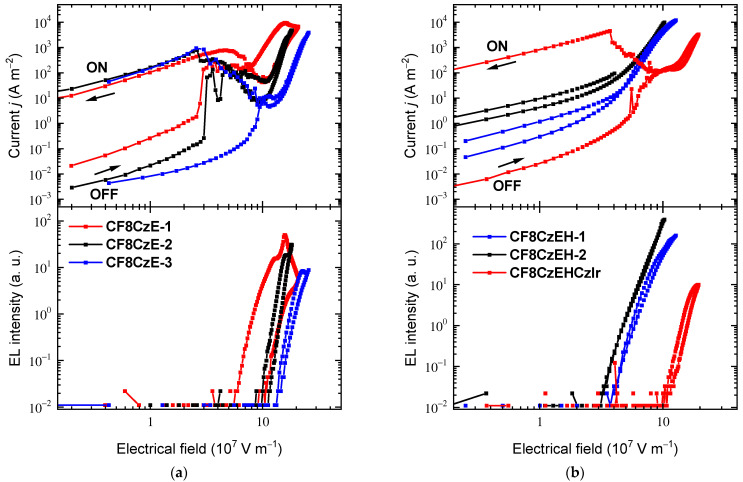
Dependence of current density and EL intensity (**a**,**b**) on applied electrical field of the ITO/PEDOT:PSS/copolymer/Ca/Al LEDs made of copolymers with thinner active layers (80–100 nm).

The LEDs made of CF8CzEH, i.e., copolymers with 2-ethylhexyl as *N*-carbazole substituent, exhibited the best performance with the lowest EL onset voltages at about 3–4 V for the active layer thicknesses of 80–252 nm, i.e., at low electrical fields. LEDs made of thicker CF8CzEH-2 active layers exhibited the best EL external quantum efficiency values up to 2%. It should be noticed that simple single-layer LEDs were investigated only. The efficiency of the LEDs could be further improved by active layer and device architecture optimization [53,54,55,56,57,58,59]. An example of the typical dependences of the current and EL intensity on the applied electrical field measured on the ITO/PEDOT:PSS/CF8CzEH/Ca/Al LEDs made of CF8CzEH-1 and CF8CzEH-2 with thicker active layers is shown in Figure 6b. The EL onset was at lower electrical field (*F* ~ 2 × 10^7^ V m^−1^) for thicker active layers than that *F* ~ 3–5 × 10^7^ V m^−1^ for the thinner ones (see Figure 7b, where the characteristics for LEDs with thinner active layers at about 100 nm are displayed). The EL efficiency of LEDs with thinner active layers was lower due to the higher currents flowing though the samples. The LEDs made of CF8CzE copolymers and CF8CzEHCzIr exhibited lower EL efficiency and anomalous behavior in their current voltage characteristics was observed as shown in Figure 7. The onset voltages of the CF8CzE LEDs were at about 6–10 V, i.e., at electrical field *F* ~ 5 × 10^7^ V m^−1^ for thicker active layers and up to *F* ~ 1 × 10^8^ V m^−1^ for thinner ones. Similarly as for the CF8CzEH LEDs, the EL efficiency was lower for the CF8CzE LEDs with thinner active layers. A steep increase in EL occurred at the onset voltage associated with the steep current increase. EL intensity saturation occurred at high current densities followed by hysteresis which indicates influence of charge trapping and/or non-radiative recombination processes. Anomalous behavior in the current-voltage characteristics of LEDs with thinner active layers about 100 nm shows a reversible memory switching phenomenon, i.e., switching between a low (ON) and high resistance (OFF) state at low voltages depending on the history of the applied voltage [48,55]. The low resistance (high conductivity) ON-state is switched to the high resistance (low conductive) OFF-state by applying a short pulse of a suitable voltage, which is higher than the position of the current maximum, e.g., 10 V. The more pronounced effect was found for the LEDs made of CF8CzE-3 and CF8CzEHCzIr, where the current difference between ON and OFF states reached four-five orders of magnitude. Such materials with anomalous behavior and memory effects are of interest in the field of bio-inspired electronics for artificial synapsis including synapses that respond to physical stimuli such as light, electric or magnetic fields, pressure and temperature or those that can detect chemicals [60,61,62].

## 4. Conclusions

Carbazole-fluorene conjugated copolymers CF8CzE, CF8CzA, CF8CzEH and CF8CzEHCzIr with various substituents in *N*-carbazole position 2-methoxycarbonylethyl, 2-carboxyethyl, 2-ethylhexyl, and nonan-2,4-dionatoiridium(III)bis(2-phenylpyridine-*N*,*C*^2′^)-9-yl, respectively, were prepared by Suzuki coupling under different conditions yielding copolymers with various average molecular weights. Effects of molecular weight and substituents attached to carbazole unit on their properties were revealed. In dilute THF solutions, the carbazole-fluorene copolymers exhibited an intense PL emission in blue spectral region of similar spectral shapes with high PL quantum yields except for the CF8CzEHCzIr copolymer with iridium complex. More pronounced differences were found in PL of the thin films, which spectra are red shifted, and EL spectra, where aggregation plays a crucial role. It was found that different centers dominate in the PL and EL processes. LEDs made of CF8CzEH copolymers with 2-ethylhexyl *N*-carbazole substituent showed efficient blue-white EL emission of the best performances with highest EL efficiency and low onset voltages. The LEDs made of CF8CzE copolymers with 2-methoxycarbonylethyl and CF8CzEHCzIr exhibited lower EL efficiency, but they showed an interesting anomalous current-voltage behavior with memory effects that were more pronounced in LEDs made of CF8CzE-3 copolymer possessing the lowest molecular weight and of CF8CzEHCzIr copolymer with the iridium complex. Such behavior and effects could be also of interest for future photonic and bio-inspired electronic applications.

## Figures and Tables

**Table 1:** Temperature, time and yield of Suzuki coupling (catalyst Pd[Ph_3_P]_4_) performed in xylene/aq.base solutions for fluorene-carbazole copolymers (*T* is temperature, *t* is time).

Copolymer	R	*T* [°C]	*t* [h]	Base	Yield [%]
CF8CzE-1	CH_2_CH_2_COOCH_3_	90	64	NaHCO_3_	82
CF8CzE-2	CH_2_CH_2_COOCH_3_	145	1.00	NaOH	86
CF8CzE-3	CH_2_CH_2_COOCH_3_	145	1.33	NaHCO_3_	84
CF8CzA ^a^	CH_2_CH_2_COOH	85	5	NaOH	78
CF8CzEH-1	2-ethylhexyl	150	1.50	NaHCO_3_	19 ^b^
CF8CzEH-2	2-ethylhexyl	150	1.50	NaHCO_3_	58 ^b^

^a^ Data for ester hydrolysis of CF8CzE-1 to CF8CzA in THF/methanol/aq.NaOH; ^b^ Yields after fractional precipitation of CF8CzEH—see Experimental.

**Table 2:** SEC data ^a^ measured in tetrahydrofuran (THF) or 1,2-dichlorobenzene (DCB) of copolymers under study (*M*_w_ is the weight-average molecular weight, *M*_n_ is the number-average molecular weight, *Ð* is the dispersity).

Copolymer	Solvent	*M*_w_	*M*_n_	*Ð*
CF8CzE-1	THF	18,500	8400	2.20
CF8CzE-2	DCB	12,310	6040	2.04
CF8CzE-3	DCB	6270	3240	1.94
CF8CzA ^b^	-	17,900	8100	2.20
CF8CzEH-1	DCB	36,080	20,680	1.74
CF8CzEH-2	DCB	49,360	34,120	1.45
CF8CzEHCzIr	DCB	16,730	9240	1.81

^a^ Polystyrene standards were used for calibration; ^b^ Calculated values considering molar mass of the structural unit.

**Table 3:** Photophysical properties of carbazole units and monomers measured in diluted THF solutions (*λ*_absmax_ absorption maximum, *λ*_PLmax_ PL maximum).

Carbazole Units and Monomers	*λ*_absmax_ (nm)	*λ*_PLmax_ ^a^ (nm)
CzE	236, 260, 294, 328, 343	347, 362
CzEH	237, 264, 295, 331, 346	349, 366
CzIr	237, 262, 295, 331, 346	349, 364, 525
CzE-Br	243, 267, 306, 331, 344	349, 367
CzEH-Br	244, 269, 306, 334, 347	352, 368
CzIr-Br	244, 267, 306, 334, 347	387, 413, 525

^a^ Excitation wavelength was *λ_abs_*_max._

**Table 4:** Photophysical properties of copolymers under study measured in dilute THF solutions (*λ*_absmax_ absorption maximum_,_ *λ*_PLmax_ PL maximum, *λ*_PLexcmax_ maximum of excitation spectra, *η*_PL_ PL quantum yield).

Copolymer	*λ*_absmax_ (nm)	*λ*_PLmax_ ^a^ (nm)	*λ*_PLexcmax_ ^b^ (nm)	*η*_PL_ ^c^
CF8CzE-1	263, 389	417, 441	381, 390	0.78
CF8CzA	268, 390	423, 445sh	268, 389	0.77
CF8CzE-2	267, 388	417, 441	266, 388	0.73
CF8CzE-3	266, 383	416, 441	265, 383	0.75
CF8CzEH-1	272, 390	419, 442	273, 385	0.87
CF8CzEH-2	273, 392	419, 441	275, 386	0.81
CF8CzEHCzIr	271, 388	418, 441	380, 388	0.23

^a^ Excitation wavelength was *λ*_absmax_, ^b^ Emission wavelength was *λ*_PLmax_, ^c^ Excitation wavelength was 360 nm.

**Table 5:** Photophysical properties of copolymers under study measured in thin films (*λ*_absmax_ absorption maximum, *λ*_PLmax_ PL maximum, *λ*_PLexcmax_ maximum of excitation spectra, *Φ*_PLrel_ relative PL efficiency).

Copolymer	*λ*_absmax_ (nm)	*λ*_PLmax_ ^a^ (nm)	*λ*_PLexcmax_ ^b^ (nm)	*ϕ*_PLrel_
CF8CzE-1	201, 245, 265, 383	427, 441	267, 297, 398	0.8
CF8CzA	204, 243, 265, 379	426, 449	265, 297, 397	0.3
CF8CzE-2	201, 245, 267, 383	431, 449	267, 297, 399	0.6
CF8CzE-3	199, 244, 264, 378	427, 450	261, 298, 398	0.2
CF8CzEH-1	203, 244, 270, 389	433, 455	265, 298, 386	1.0
CF8CzEH-2	204, 243, 271, 384	433, 454	267, 298, 387	1.0
CF8CzEHCzIr	201, 243, 270, 383	433, 452	379, 389, 399	0.05

^a^ Excitation wavelength was *λ*_absmax_, ^b^ Emission wavelength was *λ*_PLmax_.

**Table 6:** Electronic properties of the copolymers (*E*_IP_, ionization potential; *E*_A_, electron affinity; *E*_g_^elc^, electrochemical bandgap; *E*_g_^opt^, optical bandgap).

Copolymer	*E*_IP_ (eV) (−*E*_HOMO_)	*E*_A_ (eV) (−*E*_LUMO_)	*E*_g_^elc^ (eV)	*E*_g_^opt^ (eV)
CF8CzE-1	^a^ 5.24/^b^ 5.58	2.31	^a^ 2.93/^b^ 3.27	2.94
CF8CzE-2	^a^ 5.26/^b^ 5.55	2.32	^a^ 2.94/^b^ 3.23	2.94
CF8CzE-3	^a^ 5.25/^b^ 5.51	2.31	^a^ 2.94/^b^ 3.20	2.96
CF8CzEH-1	^a^ 5.23/^b^ 5.63	2.25	^a^ 2.98/^b^ 3.38	2.94
CF8CzEH-2	^a^ 5.21/^b^ 5.62	2.25	^a^ 2.96/^b^ 3.37	2.95
CF8CzEHCzIr	^a^ 5.29/^b^ 5.53	2.25	^a^ 3.04/^b^ 3.28	2.95

Evaluated from the ^a^ first and ^b^ second oxidation peak onset.

## Data Availability

Not applicable.

## Supplementary Materials

The following supporting information can be downloaded at: https://www.mdpi.com/article/10.3390/polym15132932/s1, Syntheses of monomers; Figure S1: ^1^H NMR (300.13 MHz, THF-*d*_8_, 330 K) spectrum of copolymer CF8CzE-1. Figure S2: ^13^C NMR (75.45 MHz, THF-*d*_8_, 330 K) spectrum of copolymer CF8CzE-1.; Figure S3: FTIR (ATR) spectrum of copolymer CF8CzE-1.; Figure S4: ^1^H NMR (300.13 MHz, THF-*d*_8_, 330 K) spectrum of copolymer CF8CzE-2.; Figure S5: ^13^C NMR (75.45 MHz, THF-*d*_8_, 296 K) spectrum of copolymer CF8CzE-2.; Figure S6: FTIR (ATR) spectrum of copolymer CF8CzE-2.; Figure S7: ^1^H NMR (300.13 MHz, THF-*d*_8_, 330 K) spectrum of copolymer CF8CzE-3.; Figure S8: ^13^C NMR (75.45 MHz, THF-*d*_8_, 330 K) spectrum of copolymer CF8CzE-3.; Figure S9: FTIR (ATR) spectrum of copolymer CF8CzE-3.; Figure S10: ^1^H NMR (300.13 MHz, THF-*d*_8_, 296 K) spectrum of copolymer CF8CzA.; Figure S11: FTIR (ATR) spectrum of copolymer CF8CzA.; Figure S12: ^1^H NMR (300.13 MHz, CDCl_3_, 296 K) spectra coincide for copolymers CF8CzEH-1 and CF8CzEH-2.; Figure S13: ^13^C NMR (75.45 MHz, CDCl_3_, 296 K) spectra coincide for copolymers CF8CzEH-1 and CF8CzEH-2.; Figure S14: FTIR (ATR) spectra coincide for copolymers CF8CzEH-1 and CF8CzEH-2.; Figure S15: ^1^H NMR (300.13 MHz, CDCl_3_, 296 K) spectrum of copolymer CF8CzEHCzIr.; Figure S16: ^13^C NMR (75.45 MHz, CDCl_3_, 296 K) spectrum of copolymer CF8CzEHCzIr.; Figure S17: FTIR (ATR) spectrum of copolymer CF8CzEHCzIr.; Figure S18: ^1^H NMR (300.13 MHz, THF-*d*_8_, 296 K) spectrum of methyl 3-(2,7-dibromocarbazole-9-yl)propionate.; Figure S19: FTIR (ATR) spectrum of methyl 3-(2,7-dibromocarbazole-9-yl)propionate.; Figure S20: ^1^H NMR (300.13 MHz, CDCl_3_, 296 K) spectrum of 9-(4-bromobutyl)-2,7-dibromocarbazole.; Figure S21: ^13^C NMR (75.45 MHz, CDCl_3_, 296 K) spectrum of 9-(4-bromobutyl)-2,7-dibromocarbazole.; Figure S22: FTIR (ATR) spectrum of 9-(4-bromobutyl)-2,7-dibromocarbazole.; Figure S23: ^1^H NMR (300.13 MHz, CDCl_3_, 296 K) spectrum of 9-(2,7-dibromocarbazol-9-yl)nonan-2,4-dione.; Figure S24: ^13^C NMR (75.45 MHz, CDCl_3_, 296 K) spectrum of 9-(2,7-dibromocarbazol-9-yl)nonan-2,4-dione.; Figure S25: ^1^H NMR (300.13 MHz, CDCl_3_, 296 K) spectrum of 2,7-dibromo-9-[nonan-2,4-dionatoiridium(III)bis(2-phenylpyridine-*N*,*C*^2′^)-9-yl]carbazole.; Figure S26: ^13^C NMR (75.45 MHz, CDCl_3_, 296 K) spectrum of 2,7-dibromo-9-[nonan-2,4-dionatoiridium(III)bis(2-phenylpyridine-*N*,*C*^2′^)-9-yl]carbazole.
