# Supplementary material for: Carbazole-Fluorene Copolymers with Various Substituents at the Carbazole Nitrogen: Structure—Properties Relationship
Source: Polymers (Basel). 2023 Jul 3;15(13):2932. doi: 10.3390/polym15132932 (PMC10346835; doi:10.3390/polym15132932)
Supplement: Supplementary file 1 [file polymers-15-02932-s001.zip › polymers-2464973-supplementary.pdf]

Syntheses of Monomers

Methyl 3-(2,7-dibromocarbazole-9-yl)propionate. 2,7-Dibromocarbazole (0.93 g, 2.86 mmol) and methyl acrylate (30 mL, 0.33 mol) was cooled (0–5 °C) and benzyltrimethylammonium hydroxide (0.2 m′ of 35 % methanolic solution) was added. The temperature increased slightly (ca. 2 °C) and a white crystalline material precipitated out of the solution. The starting 2,7-dibromocarbazole was not detected (TLC in toluene) in the reaction mixture after 1 h. The excess of methyl acrylate was vacuum evaporated and the residue was crystallized from methanol. Yield: 1.07 g (91 %), m. p. 137–138 °C. Anal. Calcd. for $C_{16}H_{13}Br_2NO_2$ (411.09): C 46.75, H 3.19, N 3.41, Br 38.87. Found: C 46.78, H 3.22, N 3.40, Br 38.59. ^{1}H NMR (300.13 MHz, 296 K, THF-d_8, δ): 7.95 (d, *J* = 6.0 Hz, 2H, aromatic H), 7.77 (s, 2H, aromatic H), 7.31 (d, *J* = 6.0 Hz, 2H, aromatic H), 4.62 (t, *J* = 7.1 Hz, 2H, N–CH_2), 3.57 (s, 3H, CH_3), 2.82 (t, *J* = 7.1 Hz, 2H, CH_2–CO); **(Figure S18)**. FTIR (ATR): 3073, 2993, 2953, 2842, 1729, 1583, 1480, 1450, 1419, 1356, 1325, 1300, 1259, 1208, 1162, 1058, 997, 955, 884, 799, 754, 704, 666, 601, 589, 556, 459 cm^{-1}; (**Figure S19).**

9-(4-Bromobutyl)-2,7-dibromocarbazole. 2,7-Dibromocarbazole (3.25 g, 10 mmol), 1,4-dibromobutane (27.36 g, 12.7 mmol), and tetrabutylammonium bromide (1.0 g) were vigorously stirred and heated (45 °C, 6 h) in aqueous 50% NaOH/toluene (50 mL/50 mL) bi-phase mixture under argon. Then the TLC in toluene/heptane (1:1 v/v) showed the complete consumption of 2,7-dibromocarbazole (R_F = 0.53) vs. a new spot of a product (R_F = 0.73). Toluene layer was separated and the water layer was washed with toluene (3 × 30 mL). Toluene layer and extracts were combined and dried with sodium sulfate. Toluene was vacuum evaporated and the excess of 1,4-dibromobutane was distilled off (70 °C/1 mbar) to get a white solid. The raw material was crystallized from ethanol and white crystals were obtained (yield: 2.95 g, 64%, m.p. 104–105 °C). Anal. Calcd. for $C_{16}H_{14}Br_3N$ (460.00): C 41.78, H 3.07, N 3.04, Br 52.11. Found: C 41.86, H 3.05, N 3.06, Br 52.22. ^{1}H NMR (300.13 MHz, 296 K, $CDCl_3$, δ): 7.85 (d, *J* = 8.1 Hz, 2H, aromatic H), 7.49 (s, 2H, aromatic H), 7.32 (d, *J* = 6.6 Hz, 2H, aromatic H), 4.21 (t, *J* = 6.9 Hz, 2H, N–CH_2), 3.80 (t, *J* = 6.3 Hz, 2H, CH_2–Br), 2.04–1.97 (m, 2H, CH_2), 1.93–1.84 (m, 2H, CH_2); **(Figure S20)**. ^{13}C NMR (75.45 MHz, $CDCl_3$, 296 K, δ): 141.2, 122.8, 121.6, 121.4, 119.9, 111.9 (all aromatic C), 42.5, 32.8, 30.1, 27.5 (all aliphatic C); (**Figure S21**). FTIR (ATR): 3073, 2935, 2898, 1584, 1483, 1450, 1421, 1326, 1315, 1247, 1130, 1055, 997, 919, 850, 793, 742, 666, 594, 556, 457 cm^{-1}; (**Figure S22**).

9-(2,7-Dibromocarbazol-9-yl)nonan-2,4-dione. Sodium hydride (33.4 mmol, 1.34 g of 60% NaH in mineral oil) was stirred in dry THF (85 mL) under argon and cooled to 0 °C. The acetylacetone (33.4 mmol, 3.34 g) was added dropwise by a syringe via septum and the reaction mixture was stirred 20 min forming a drift-ice matter. n-Butyllithium (33.4 mmol, 2.5 M in hexane) was added and after 20 min of mixing at 0 °C, the 9-(4-bromobutyl)-2,7-dibromocarbazole (6.68 mmol, 3.07 g) in dry THF (11 mL) was added within 3 min. The reaction mixture was stirred at 0 °C (1 h) and at room temperature (1 h) and then quenched with saturated aqueous ammonium chloride (18 mL) and acidified with 2 M HCl (29 mL). Organic phase was separated and water phase was extracted with ethyl acetate (2 × 50 mL). Combined organic phases were washed with saturated sodium chloride and water and dried with anhydrous sodium sulfate. The sodium sulfate was filtered off, solvent was vacuum evaporated and the raw material was dried (oil pump 24 h). The pure product was obtained by column chromatography (silica gel) in toluene. White (colorless) crystals, m.p. 106–107 °C, were obtained (yield: 2.07 g, 64%). R_F (TLC, silica gel) = 0.21 (toluene) or 0.77 (chloroform/butyl acetate (20:1 by vol.)). Anal. Calcd for $C_{21}H_{21}Br_2NO_2$(479.20): C, 52.63; H, 4.42; N, 2.92; Br, 33.35. Found: C, 53.01; H, 4.43; N, 2.94; Br, 33.58. ^{1}H NMR (300.13 MHz, $CDCl_3$, δ): 15.43 (s, 1H×0.850, enol hydrogen C–OH), 7.85 (d, 2H, *J* = 8.4 Hz, heterocyclic), 7.49 (d, 2H, *J* = 1.5 Hz, heterocyclic), 7.31 (dd, 2H, *J* = 8.1 and 1.6 Hz, heterocyclic), 5.41 (s, 1H×0.905, enol hydrogen C=CH–CO), 4.16 (t, 2H, *J* = 7.2 Hz, N–CH_2), 3.52 (s, 2H × 0.161, keto hydrogen CO–CH_2–CO), 2.22 (t, 2H, *J* = 7.2 Hz, CH_2–CO), 2.02 (s, 3H, CH_3), 1.83 (m, 2H, CH_2), 1.62 (m, 2H, CH_2), 1.38 (m, 2H, CH_2); (**Figure S23**). ^{13}C NMR (75.45 MHz, $CDCl_3$, δ): 193.6 (C=O), 191.4 (C=O), 141.3, 122.7, 121.6, 121.3,

119.8, 112.0 (all 2C heterocyclic), 99.9, 43.1, 38.0, 31.0, 28.6, 26.7, 25.2 (all 1C aliphatic); (**Figure S24**).

The dione can exist in a keto or enol form. From the ^{1}H NMR spectrum (Figure S23) it is clear that the enol form predominates. If we compare the integrals of signals at 15.43 (insert, enol hydrogen, C–OH, 0.850), 5.41 (enol hydrogen, C=CH–CO, 0.905), and 3.52 (keto hydrogen, CO–CH_2–CO, 0.161) ppm, we can estimate the enol form amount as much as 85–90 %. Of course, there is an equilibrium between enol and keto form, so the sum of 0.850 + 0.905 + 0.161 (= 1.916) should be theoretically equal to 2. Acceptable difference is probably due to inaccuracies in the integration of the NMR signals.

Tetrakis(2-phenylpyridine-*N*,*C*$^{2'}$)(*μ*-dichloro)diiridium. The reactive chlorine-bridged dimer was synthesized from 2-phenylpyridine and iridium trichloride hydrate according to the known procedure [40].

2,7-Dibromo-9-[nonan-2,4-dionatoiridium(III)bis(2-phenylpyridine-*N*,*C*$^{2'}$)-9-yl]-carbazole.

The 9-(2,7-dibromocarbazol-9-yl)nonan-2,4-dione (0.75 g, 1.565 mmol) and tetrakis(2-phenylpyridine-*N*,*C*$^{2'}$)(*μ*-dichloro)diiridium (0.75 g, 0.700 mmol) were heated (110 °C) in 2-ethoxyethanol (50 mL) in the presence of a base (0.75 g Na_2CO_3) for 24 h. The reaction was followed by TLC in chloroform (product: R_F = 0.21) or in chloroform/butyl acetate (20:1 v/v) (product: R_F = 0.60). The sodium carbonate was filtered off and reaction mixture was vacuum evaporated. The residue was purified by gradient column chromatography (silica gel) in chloroform → chloroform/butyl acetate (20:1 v/v). The corresponding fractions (R_F = 0.60) were collected, vacuum evaporated and dried (oil pump) to constant weight. Yellow-green powder, m.p. 124–125 °C, was obtained (yield: 0.423 g, 28%). R_F (TLC, silica gel) = 0.21 (chloroform) or 0.60 (chloroform/butyl acetate (20:1 v/v)). Anal. Calcd for $C_{43}H_{36}Br_2N_2O_3Ir$ (978.79): C, 52.77; H, 3.71; N, 4.29; Br, 16.33. Found: C, 52.57; H, 3.65; N, 4.05; Br, 16.65. ^{1}H NMR (300.13 MHz, $CDCl_3$, δ): 8.47–6.23 (m, 22H, carbazole and 2-phenylpyridine hydrogens), 5.14 (s, 1H, CO–CH–CO), 3.89–3.86 (m, 2H, N–CH_2), 2.02–1.98 (m, 2H, CO–CH_2), 1.76 (s, 3H, CH_3), 1.58–1.53 (m, 2H, CH2), 1.31 (m, 2H, CH_2), 1.05 (m, 2H, CH_2); (**Figure S25**). ^{13}C NMR (75.45 MHz, $CDCl_3$, δ): 187.2 (C=O), 185.1 (C=O), 168.7, 151.7, 147.8, 144.7, 141.3, 136.9, 133.2, 129.1, 123.9, 123.6, 122.6, 121.5, 121.3, 120.7,119.7, 118.4, 112.0 (2C all aromatic), 99.9, 43.2, 40.9, 38.0, 28.6, 26.3, 25.2 (all 1C, aliphatic); (**Figure S26**).

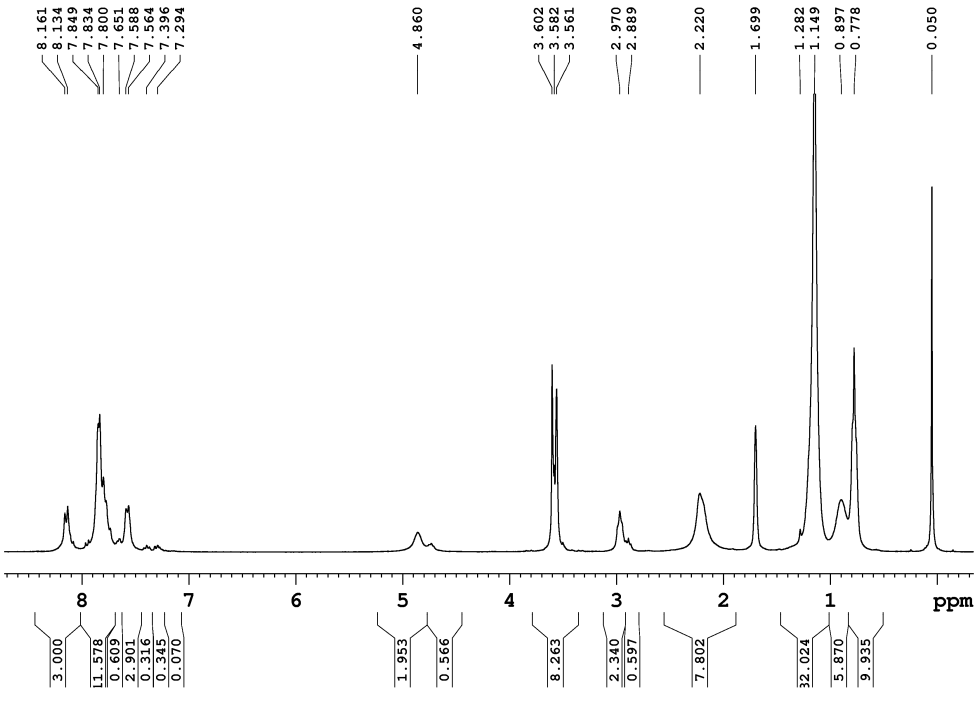

Figure S1. ^{1}H NMR (300.13 MHz, THF-*d*$_8$, 330 K) spectrum of copolymer CF8CzE-1.

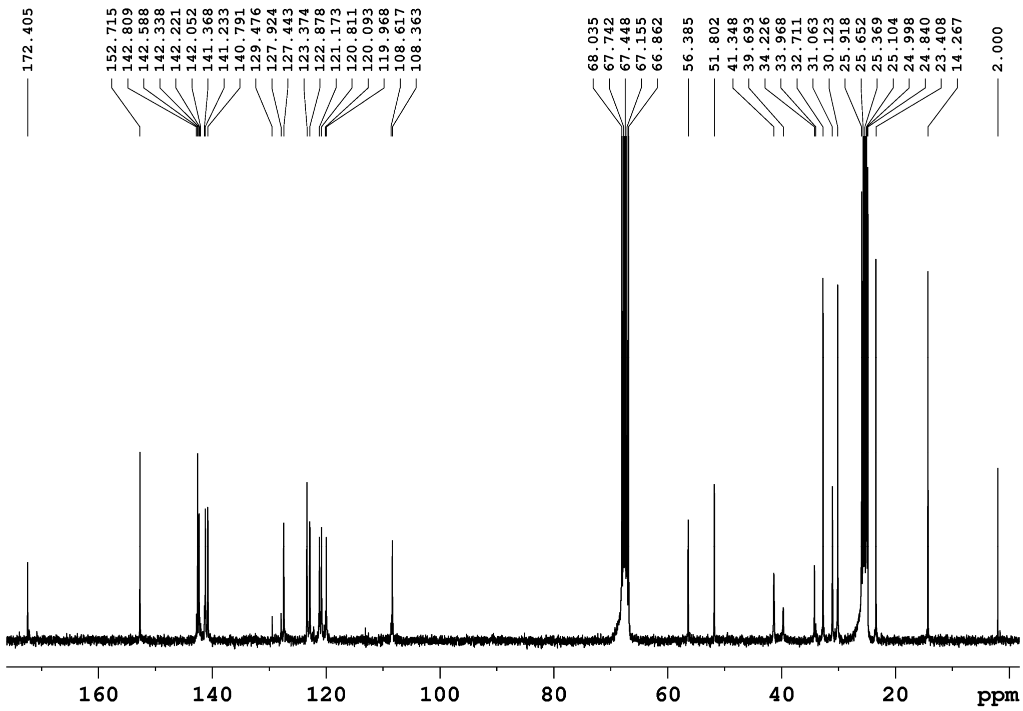

Figure S2. ^{13}C NMR (75.45 MHz, THF-d_8, 330 K) spectrum of copolymer CF8CzE-1.

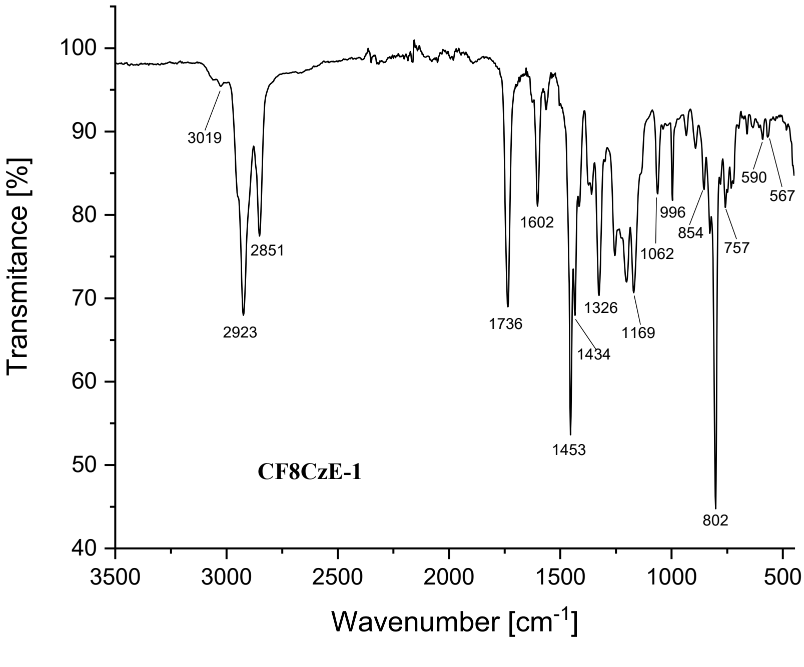

Figure S3. FTIR (ATR) spectrum of copolymer CF8CzE-1.

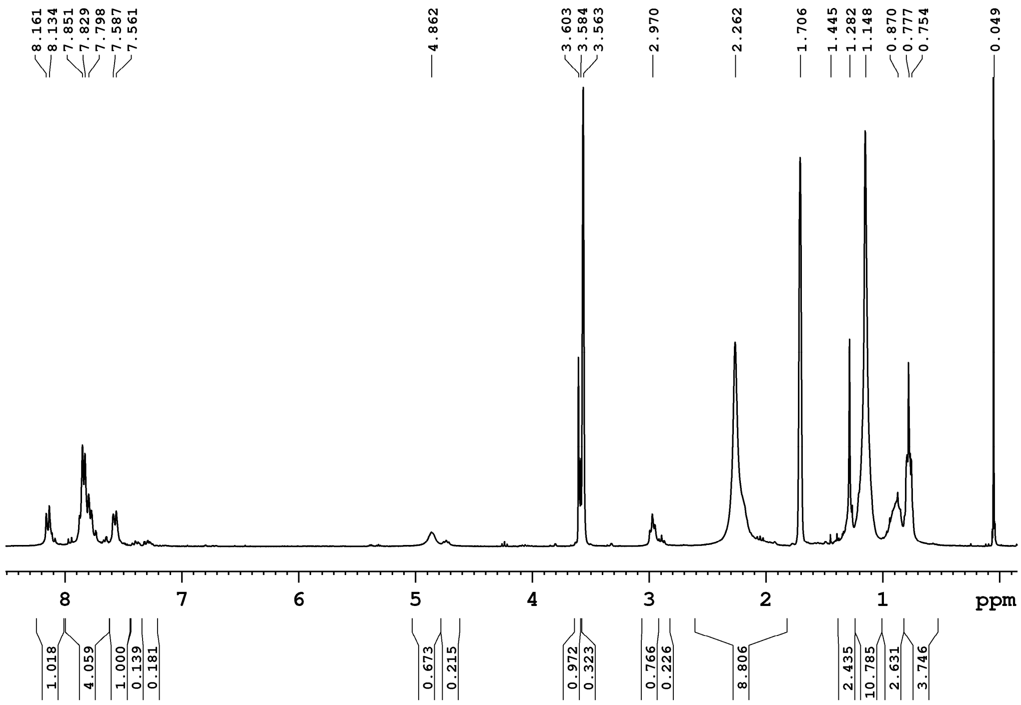

Figure S4. ^{1}H NMR (300.13 MHz, THF-*d*$_8$, 330 K) spectrum of copolymer CF8CzE-2.

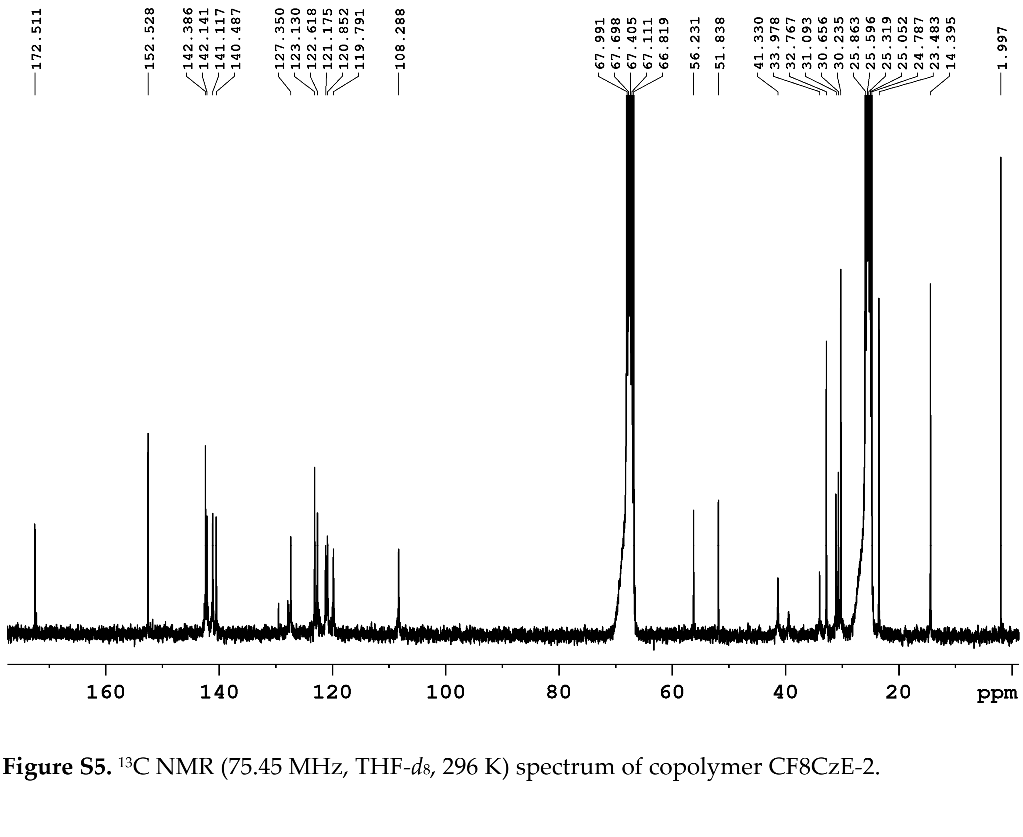

Figure S5. ^{13}C NMR (75.45 MHz, THF-*d*$_8$, 296 K) spectrum of copolymer CF8CzE-2.

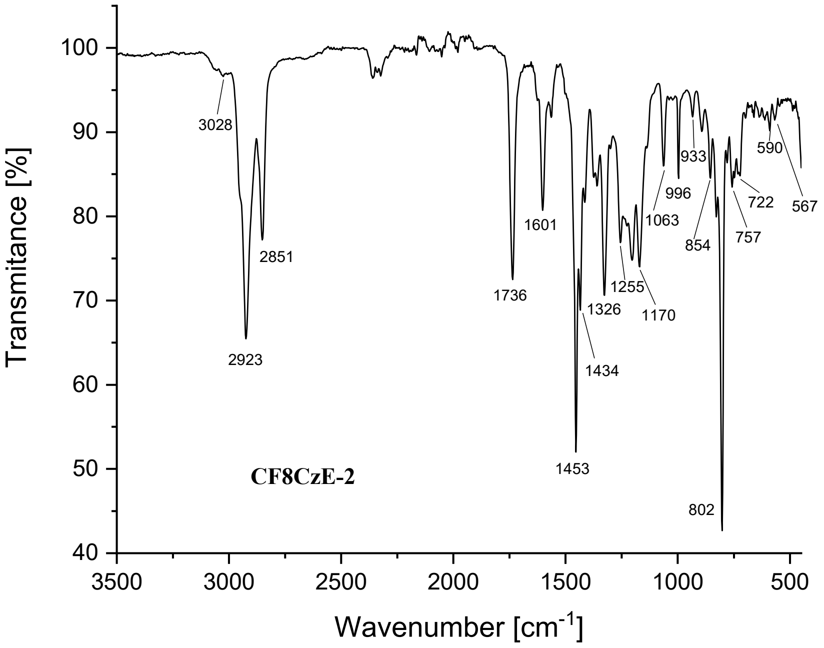

Figure S6. FTIR (ATR) spectrum of copolymer CF8CzE-2.

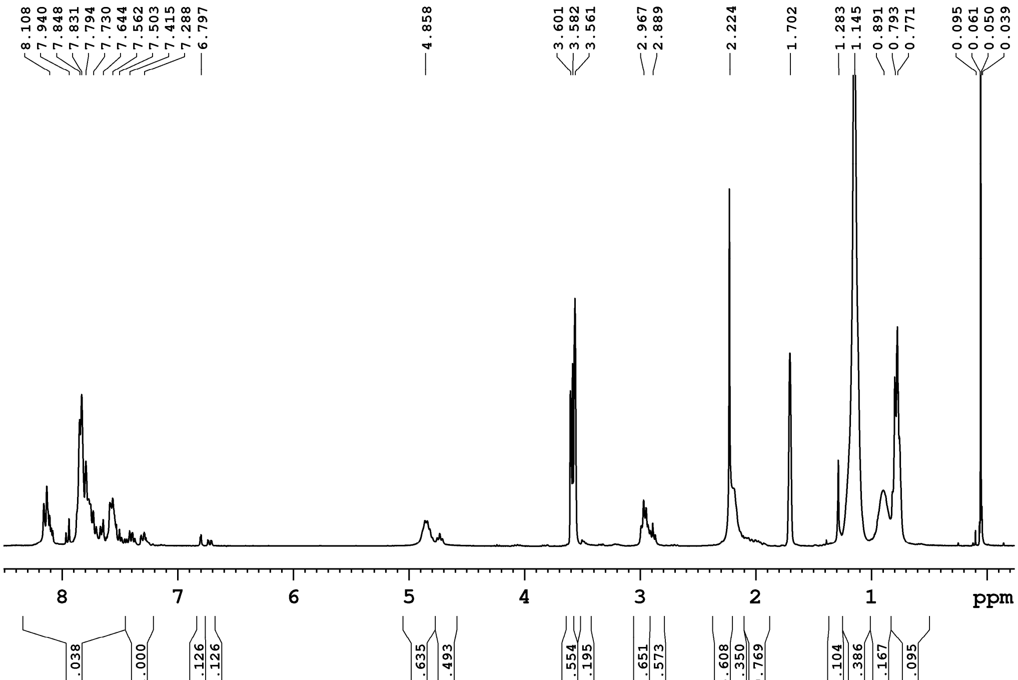

Figure S7. ^{1}H NMR (300.13 MHz, THF-d_8, 330 K) spectrum of copolymer CF8CzE-3.

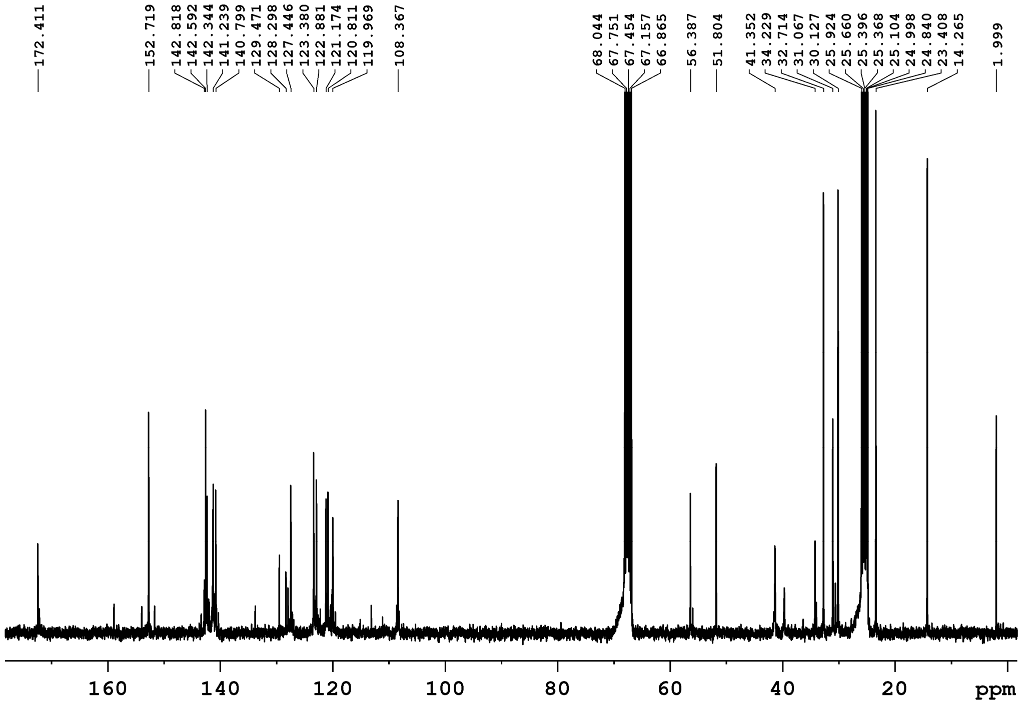

Figure S8. ^{13}C NMR (75.45 MHz, THF-d_8, 330 K) spectrum of copolymer CF8CzE-3.

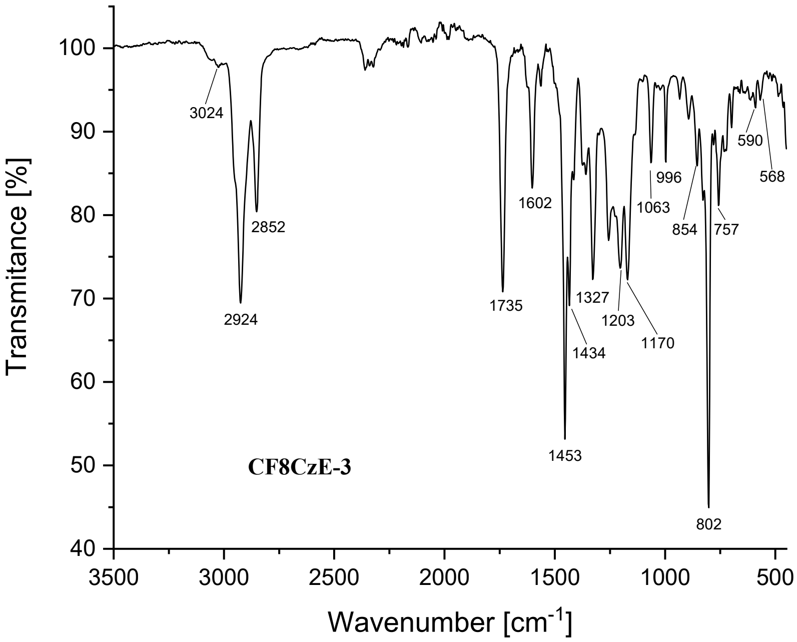

Figure S9. FTIR (ATR) spectrum of copolymer CF8CzE-3.

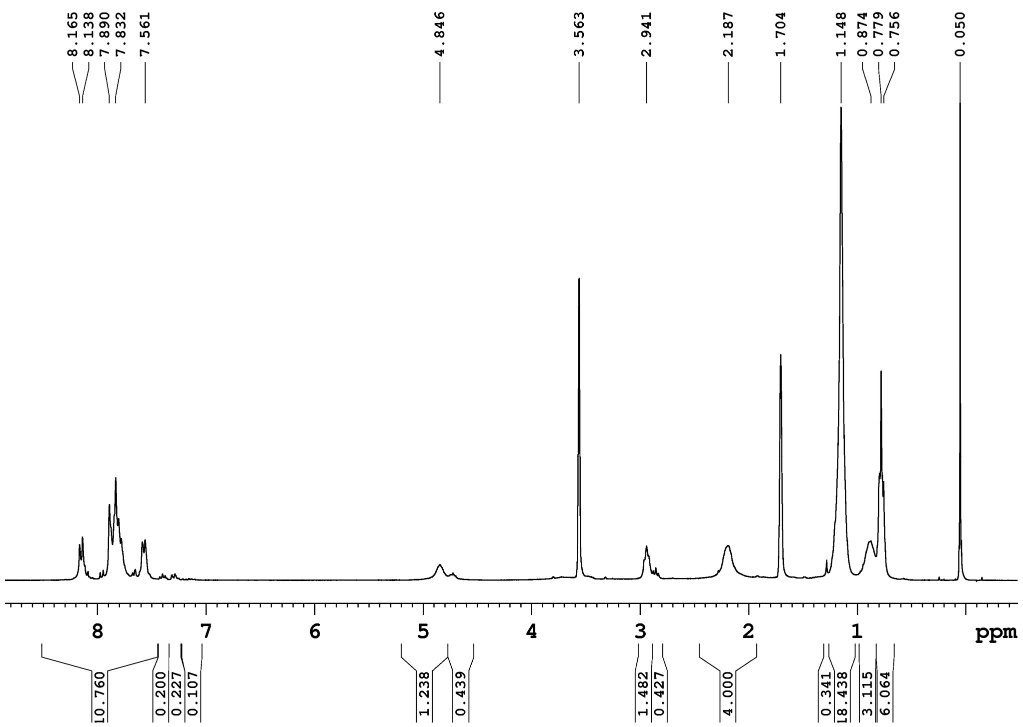

Figure S10. ^{1}H NMR (300.13 MHz, THF-d_8, 296 K) spectrum of copolymer CF8CzA.

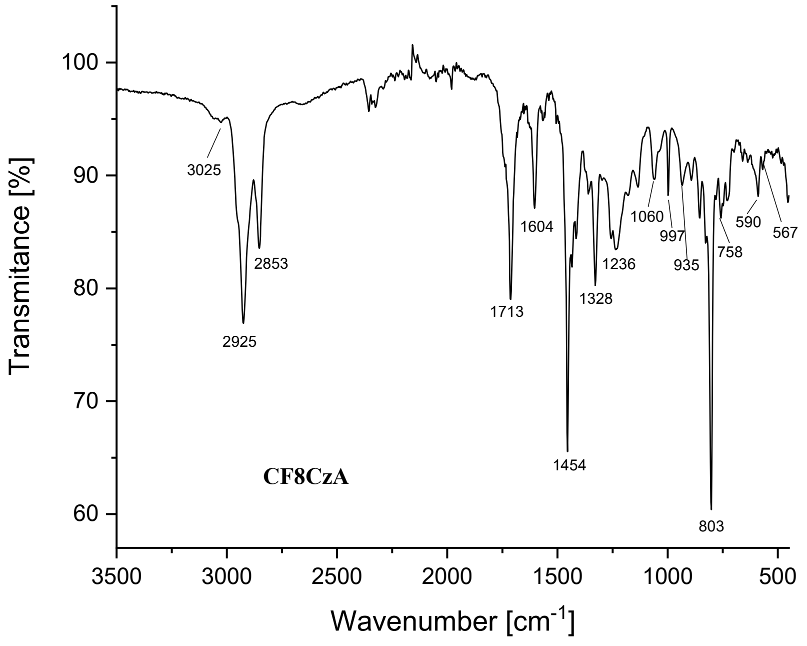

Figure S11. FTIR (ATR) spectrum of copolymer CF8CzA.

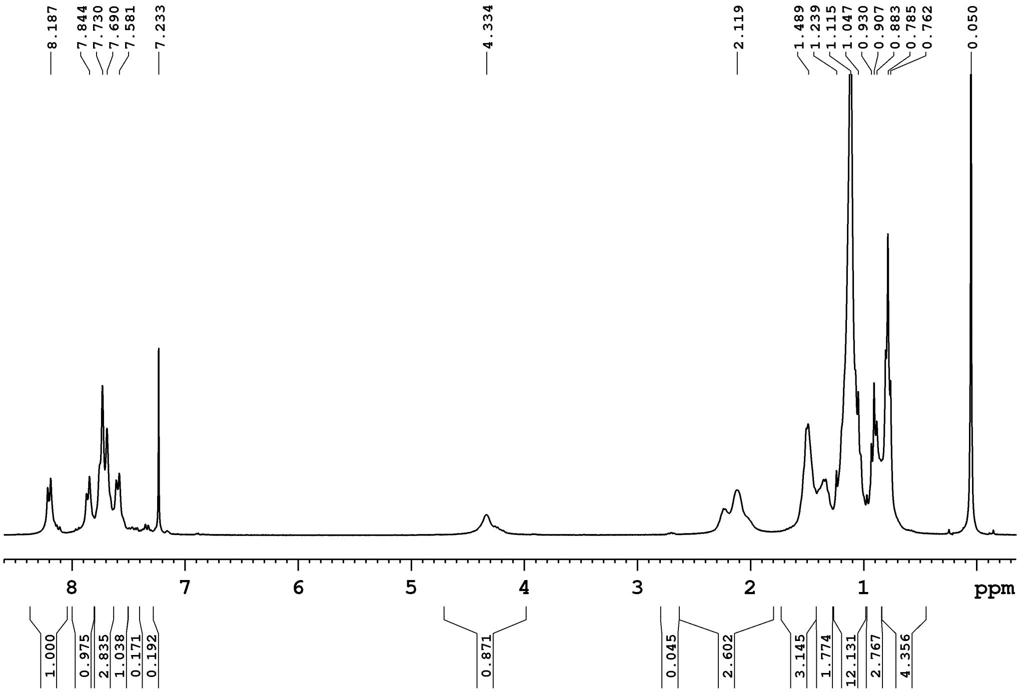

Figure S12. ^{1}H NMR (300.13 MHz, $CDCl_3$, 296 K) spectra coincide for copolymers CF8CzEH-1 and CF8CzEH-2.

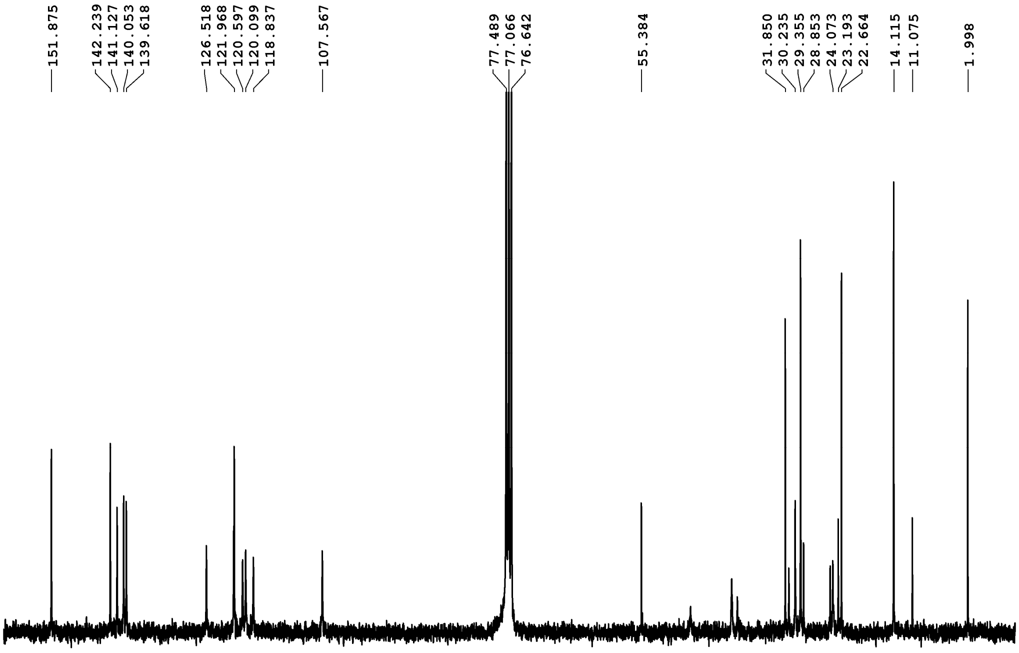

Figure S13. ^{13}C NMR (75.45 MHz, $CDCl_3$, 296 K) spectra coincide for copolymers CF8CzEH-1 and CF8CzEH-2.

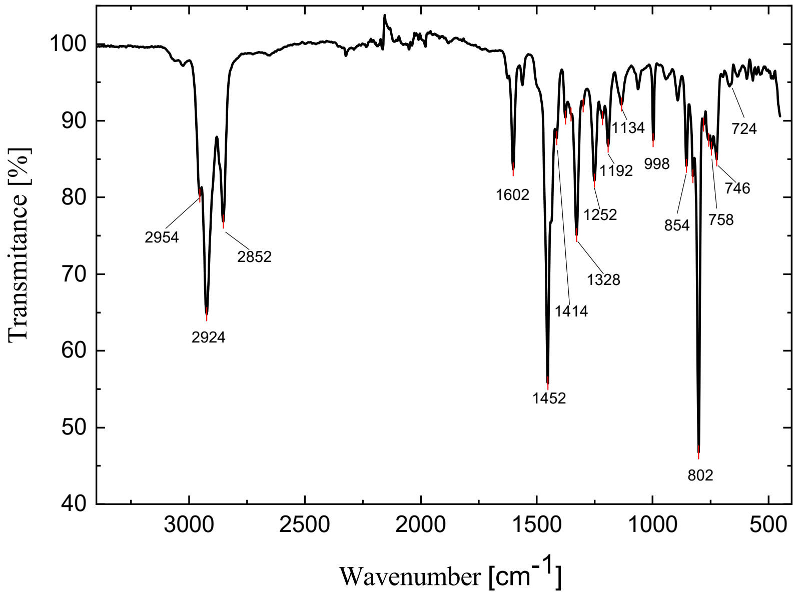

Figure S14. FTIR (ATR) spectra coincide for copolymers CF8CzEH-1 and CF8CzEH-2.

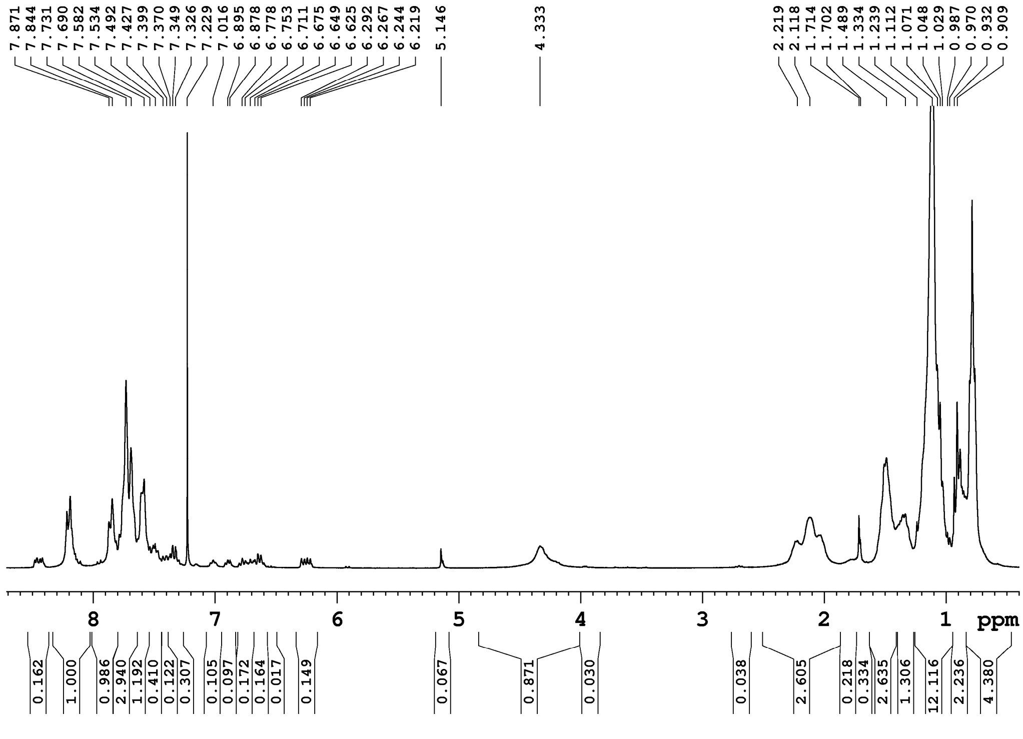

Figure S15. ^{1}H NMR (300.13 MHz, $CDCl_3$, 296 K) spectrum of copolymer CF8CzEHCzIr.

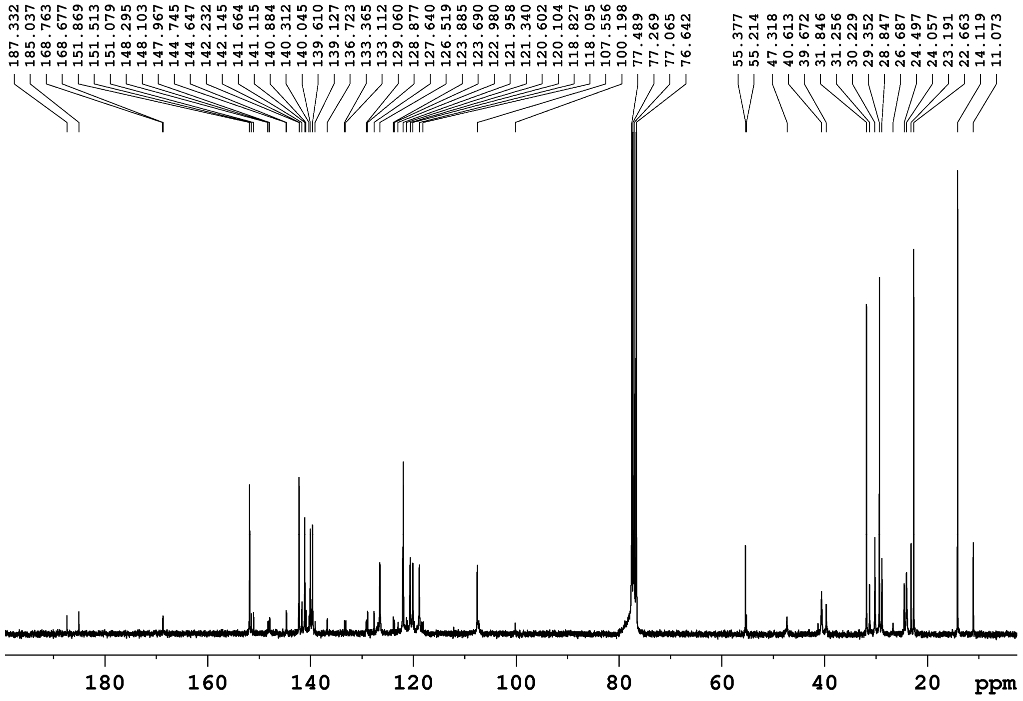

Figure S16. ^{13}C NMR (75.45 MHz, $CDCl_3$, 296 K) spectrum of copolymer CF8CzEHCzIr.

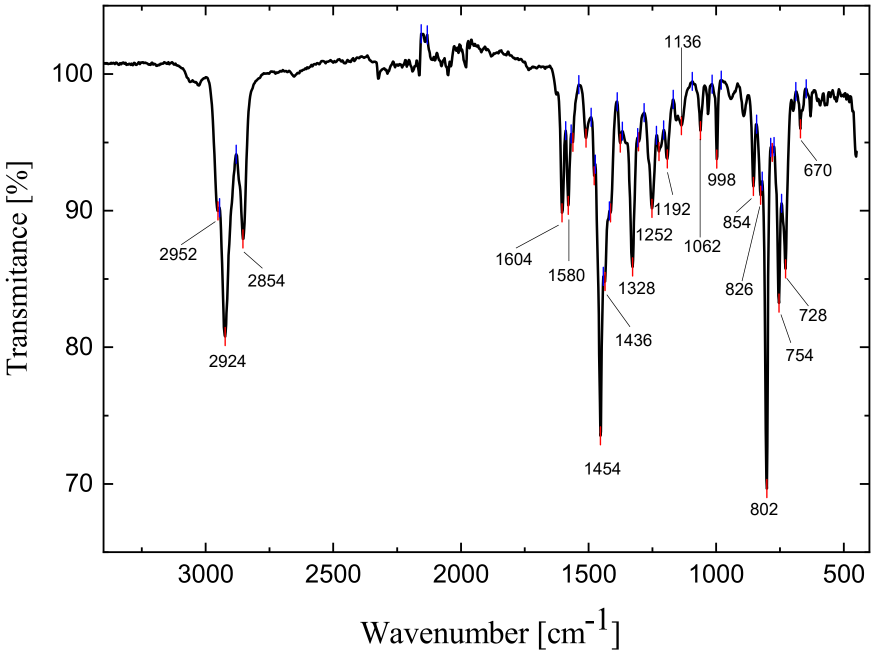

Figure S17. FTIR (ATR) spectrum of copolymer CF8CzEHCzIr.

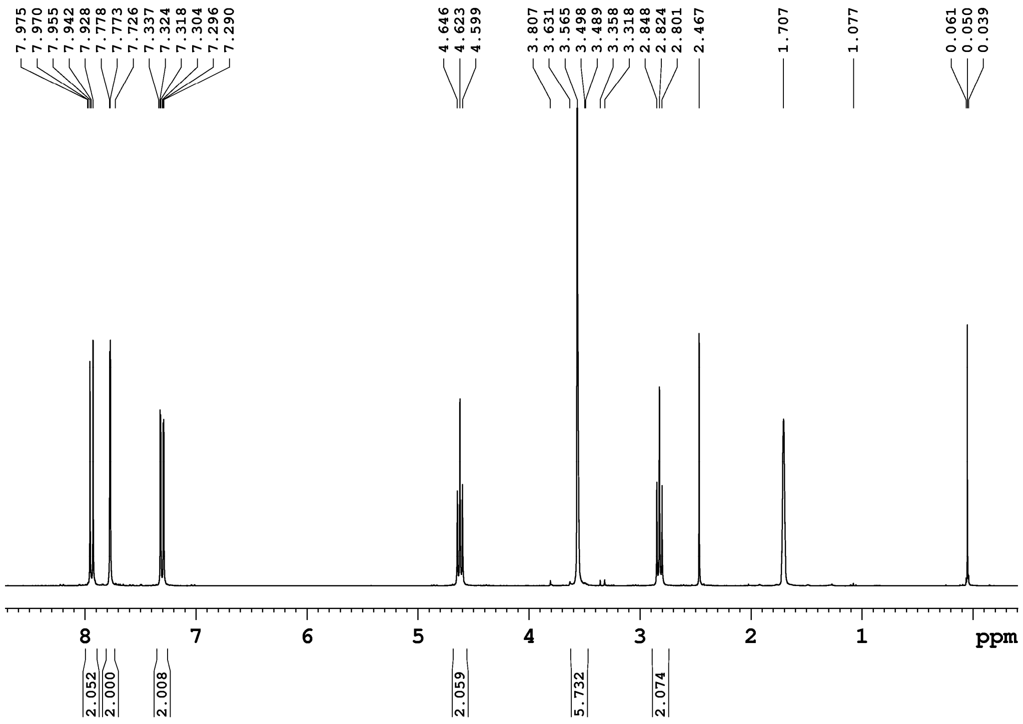

Figure S18. ^{1}H NMR (300.13 MHz, THF-d_8, 296 K) spectrum of methyl 3-(2,7-dibromocarbazole-9-yl)propionate.

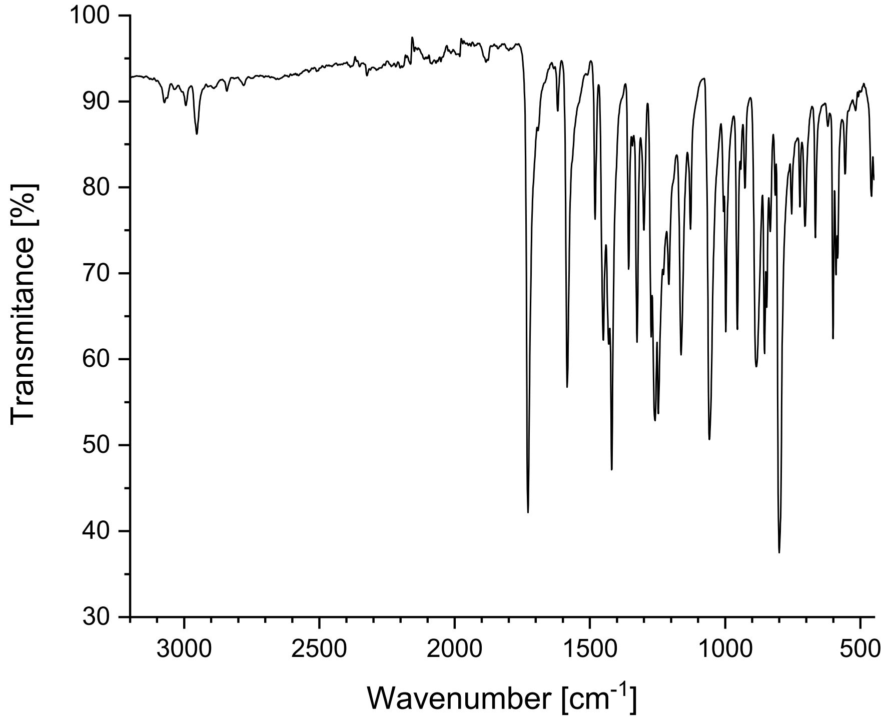

Figure S19. FTIR (ATR) spectrum of methyl 3-(2,7-dibromocarbazole-9-yl)propionate.

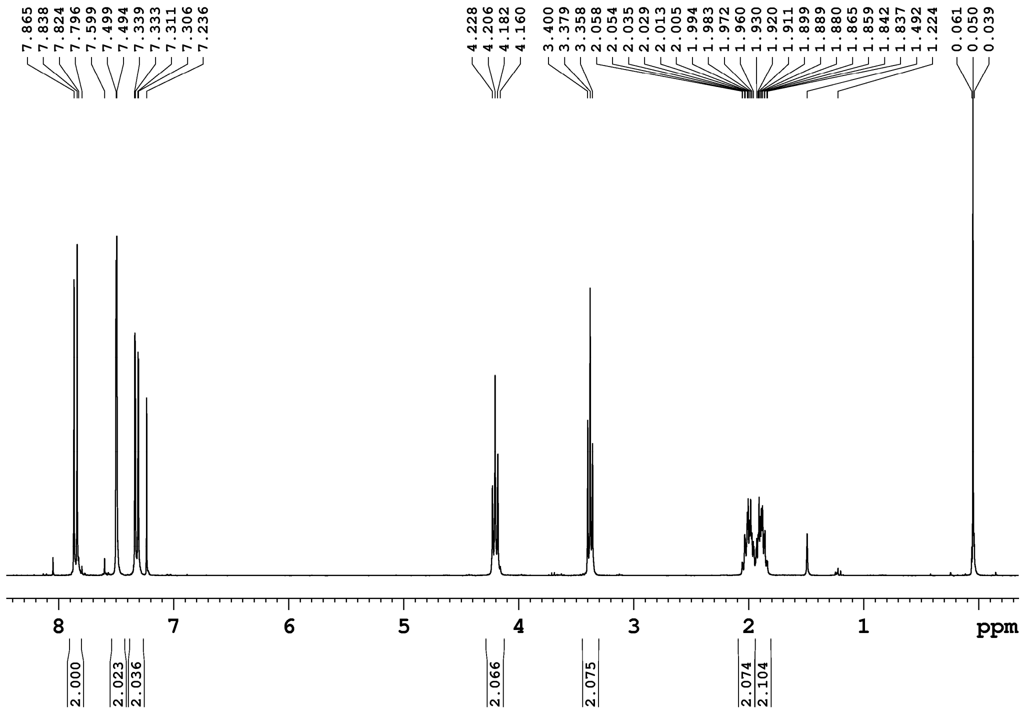

Figure S20. ^{1}H NMR (300.13 MHz, $CDCl_3$, 296 K) spectrum of 9-(4-bromobutyl)-2,7-dibromocarbazole.

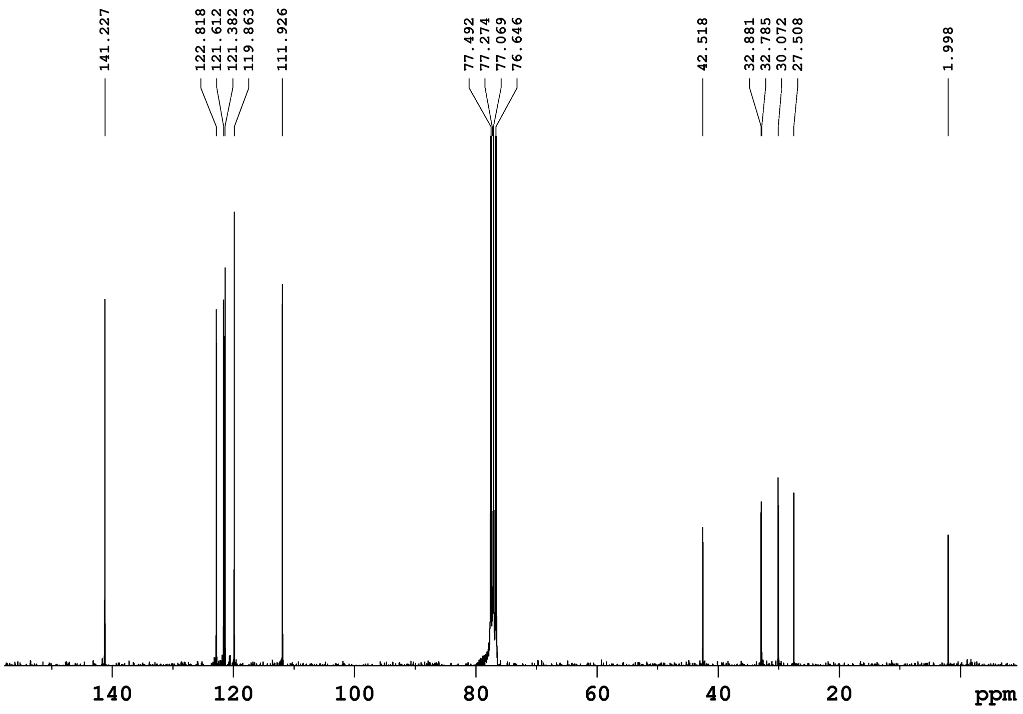

Figure S21. ^{13}C NMR (75.45 MHz, $CDCl_3$, 296 K) spectrum of 9-(4-bromobutyl)-2,7-dibromocarbazole.

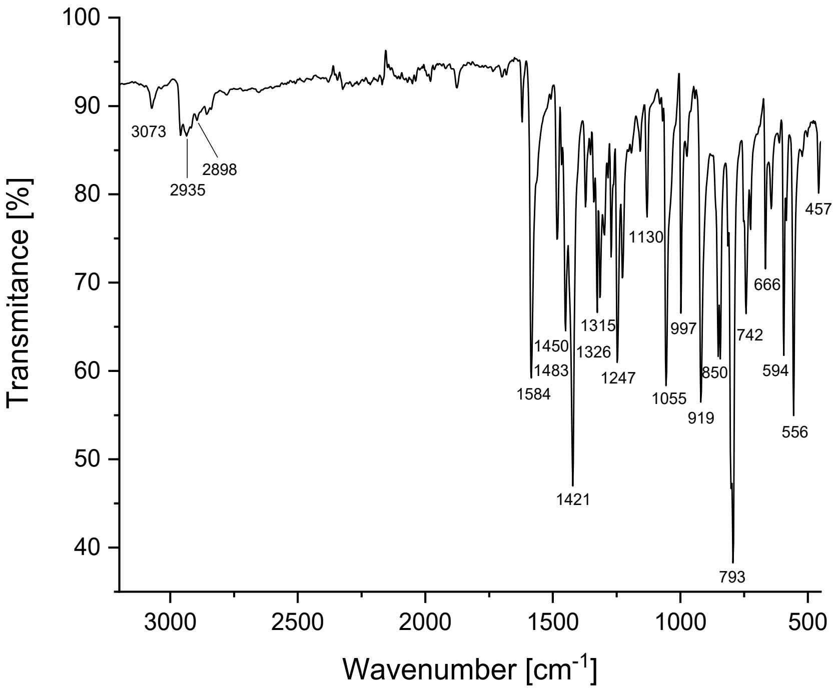

Figure S22. FTIR (ATR) spectrum of 9-(4-bromobutyl)-2,7-dibromocarbazole.

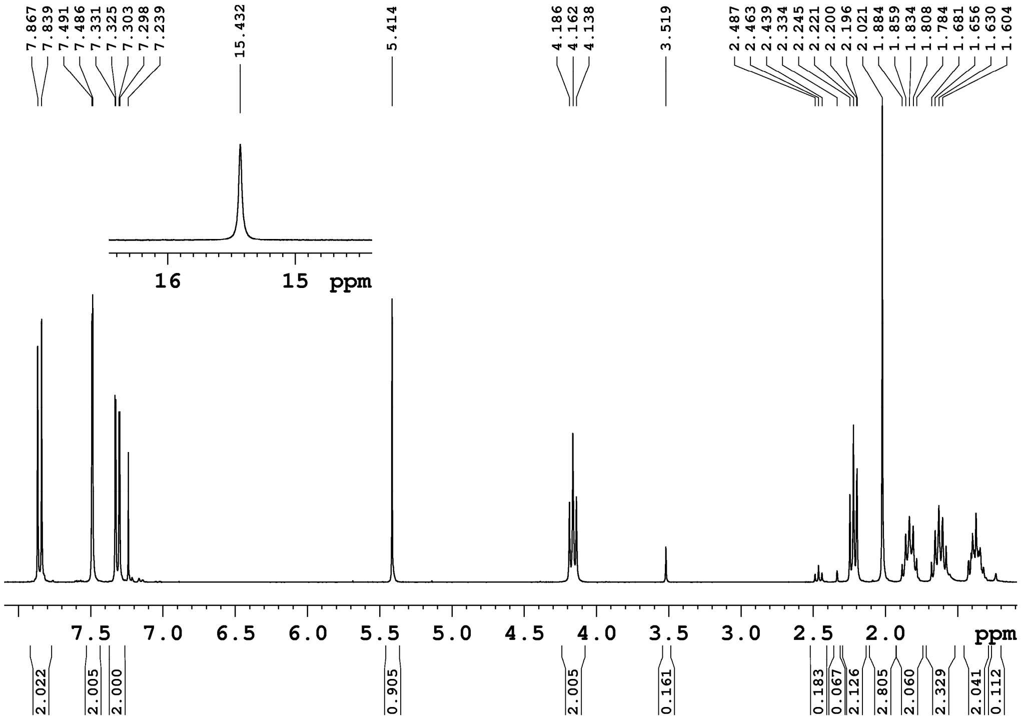

Figure S23. ^{1}H NMR (300.13 MHz, $CDCl_3$, 296 K) spectrum of 9-(2,7-dibromocarbazol-9-yl)nonan-2,4-dione.

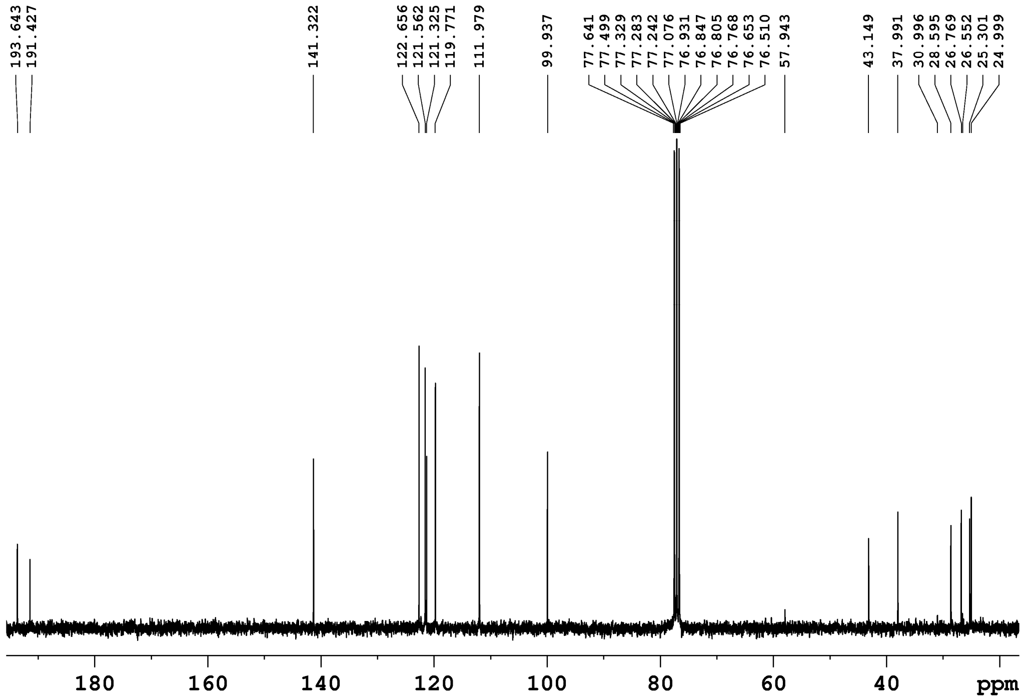

Figure S24. ^{13}C NMR (75.45 MHz, $CDCl_3$, 296 K) spectrum of 9-(2,7-dibromocarbazol-9-yl)nonan-2,4-dione.

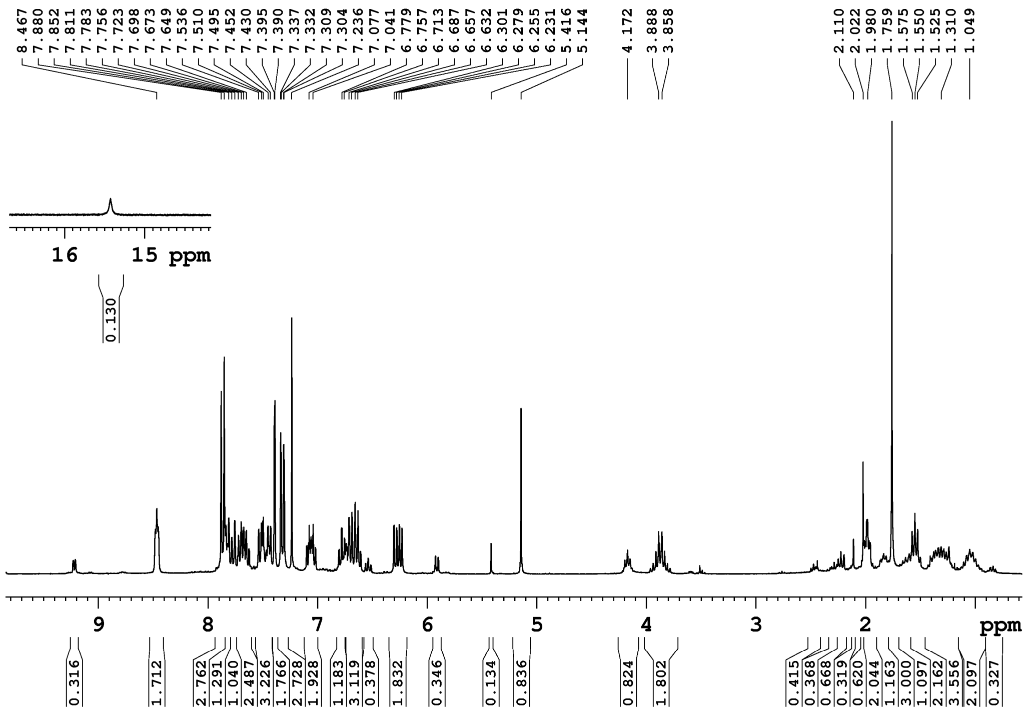

Figure S25. ^{1}H NMR (300.13 MHz, $CDCl_3$, 296 K) spectrum of 2,7-dibromo-9-[nonan-2,4-dionatoiridium(III)bis(2-phenylpyridine-*N*,*C*$^{2'}$)-9-yl]carbazole.

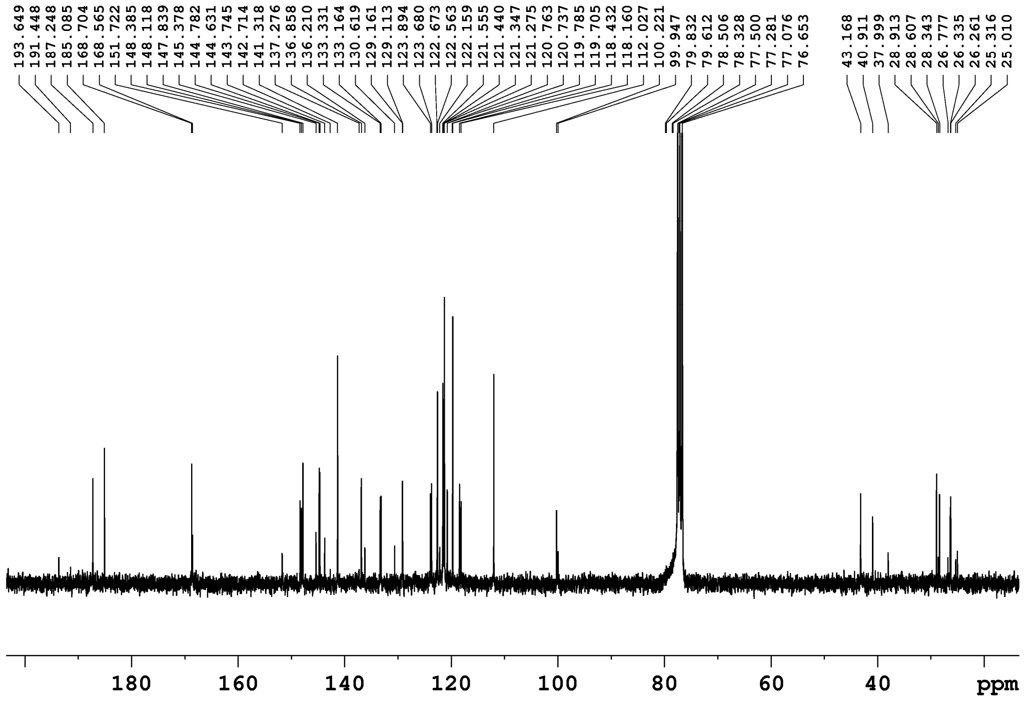

Figure S26. ^{13}C NMR (75.45 MHz, $CDCl_3$, 296 K) spectrum of 2,7-dibromo-9-[nonan-2,4-dionatoirid-ium(III)bis(2-phenylpyridine-*N*,$C^{2'}$)-9-yl]carbazole.
